# Heavy Metal Resistance in *Salmonella* Typhimurium and Its Association With Disinfectant and Antibiotic Resistance

**DOI:** 10.3389/fmicb.2021.702725

**Published:** 2021-08-04

**Authors:** Ghulam Raza Mustafa, Ke Zhao, Xueping He, Shujuan Chen, Shuliang Liu, Ahsan Mustafa, Li He, Yong Yang, Xiumei Yu, Petri Penttinen, Xiaolin Ao, Aiping Liu, Muhammad Zubair Shabbir, Xuebin Xu, Likou Zou

**Affiliations:** ^1^College of Resources, Sichuan Agricultural University, Chengdu, China; ^2^College of Food Science, Sichuan Agricultural University, Ya’an, China; ^3^Key Laboratory for Animal Disease-Resistance Nutrition of China, Ministry of Education, Institute of Animal Nutrition, Sichuan Agricultural University, Chengdu, China; ^4^Institute of Microbiology, University of Veterinary and Animal Sciences, Lahore, Pakistan; ^5^Shanghai Municipal Center for Disease Control and Prevention, Shanghai, China

**Keywords:** *Salmonella* Typhimurium, heavy metal resistance, disinfectant resistance, antibiotic resistance, heavy metal resistance gene, conjugation

## Abstract

Metals are widely used in animal feed for their growth-stimulating and antimicrobial effects, yet their use may potentially promote the proliferation of antibiotic resistance through co-selection. We studied the prevalence and associations of metal, antibiotic, and disinfectant resistances of 300 *Salmonella* Typhimurium isolates from pig meat, pig manure, chicken meat, poultry manure, and human stool from Sichuan, China. Seventy four percent of the 300 *Salmonella* Typhimurium isolates were considered resistant to Cu, almost 50% to Zn and Cr, over 25% to Mn and Cd, and almost 10% to Co. Most of the isolates carried at least one heavy metal resistance gene (HMRG). The Cr-Zn-Cd-resistance gene *czcD* was carried by 254 isolates and the Cu-resistance genes *pcoR* and *pcoC* by 196 and 179 isolates, respectively. Most of the isolates were resistant to at least one antibiotic and almost 80% were multidrug-resistant. The prevalence of resistance to six antibiotics was higher among the pig meat and manure isolates than among other isolates, and that of streptomycin and ampicillin were highest among the pig meat isolates and that of ciprofloxacin and ofloxacin among the pig manure isolates. From 55 to 79% of the isolates were considered resistant to disinfectants triclosan, trichloroisocyanuric acid, or benzalkonium chloride. The metal resistances and HMRGs were associated with resistance to antibiotics and disinfectants. Especially, Cu-resistance genes were associated with resistance to several antibiotics and disinfectants. The transfer of the Cr-Zn-Cd-resistance gene *czcD*, Cu-resistance gene *pcoC*, and Co-Ni-resistance gene *cnrA* into *Escherichia coli* and the increased Cu-resistance of the transconjugants implied that the resistance genes were located on conjugative plasmids. Thus, the excessive use of metals and disinfectants as feed additives and in animal care may have the potential to promote antibiotic resistance through co-selection and maintain and promote antibiotic resistance even in the absence of antibiotics.

## Introduction

*Salmonella* infections are a major public health concern worldwide (Sodagari et al., 2020). *Salmonella* is the second most common cause of food-borne diseases worldwide and is associated with more deaths than any other food-borne disease in the developed countries (WHO, 2020). Food-borne Disease Active Surveillance Network (Foodnet) reported *Salmonella* as the leading cause of food-borne disease-related deaths in the United States (Barton Behravesh et al., 2011). Among the food-borne infections caused by a single etiologic agent in the United States in 2015, *Salmonella* was responsible for 34% of the outbreaks, 39% of the illnesses, 64% of the hospitalizations, and 60% of the deaths (Dewey-Mattia et al., 2018). The European Food Safety Authority (EFSA) confirmed salmonellosis as the second most common zoonosis with over 90,000 confirmed cases in 2017 (EFSA and ECDC, 2018). The food-borne surveillance system in China suggested that *Salmonella* was the second most common bacteria causing food-borne outbreaks during 2000–2014 (Luo et al., 2017). Thus, the *Salmonella* infection-related mortality and morbidity burden societies worldwide (Majowicz et al., 2010). For example, in England, the *Salmonella* infections, characterized by diarrhea, fever, and abdominal cramps, leading to over 11,000 annual GP consultations (Tam et al., 2012; Barr and Smith, 2014).

More than 2,600 serotypes of *Salmonella* have been identified (Takaya et al., 2020). *Salmonella* Typhimurium is one of the predominant serotypes in many countries, including China (Ceyssens et al., 2015; Wang Y. et al., 2017; Simpson et al., 2018) and it has held first or second place in China for many years (Ran et al., 2011; Wang Y. et al., 2017). *Salmonella* Typhimurium was also reported as the most frequently isolated serotype of non-typhoidal *Salmonella* from food-borne illnesses in different provinces of China (Liang et al., 2015), which represented 25.5, 29.44, and 39.7%, of the isolates obtained from diarrheal disease surveillance between 2006-2010 in Shanghai, 2010–2014 and 2013–2018 in Shenzhen, China (Zhang et al., 2014; Li W. et al., 2017; Shen et al., 2020). *Salmonella* is associated with a wide variety of foods, yet animal products, especially pig and poultry, are the main source of *Salmonella* (Hugas and Beloeil, 2014; Antunes et al., 2016; Demirbilek, 2017; Heredia and García, 2018). The routes of transmission for *Salmonella* Typhimurium include contaminated meat, eggs, and manure (Antunes et al., 2016; Chousalkar et al., 2018; Kumar et al., 2019). With the development of China, the average intake of meat, especially pig increased from 37.1 g/day in 1992 to 64.3 g/day per person in 2012 and was expected to surpass 100 g/day people by 2020 which has indirectly increased the risk of food-borne zoonoses, including salmonellosis (He et al., 2016; CIIN, 2018a,b; Pan et al., 2018, 2019). In China, *Salmonella* Typhimurium strains are commonly isolated from retail meat, particularly pig, suggesting a link between human infections with this serotype and pig products.

**FIGURE 1:**
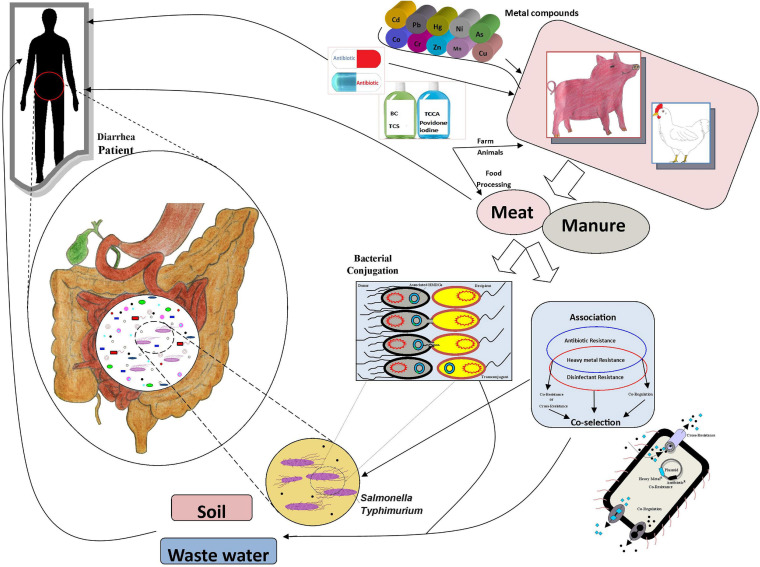
The associations and transmission routes of metal, antibiotic, and disinfectant resistance.

Antibiotics have commonly been used to treat animal diseases and as growth promoters (Allen et al., 2013). However, a substantial amount of the antibiotics are excreted in feces and enter soil via manure (Kumar et al., 2005). Due to food safety and health issues, many countries, e.g., in the European Union, have banned the use of antibiotics in the feed of animals as growth promoters (Burch, 2006). Both WHO and individual countries have introduced guidelines to withdraw medicated feed additives to combat antimicrobial resistance from animal resources (Hu and Cowling, 2020). Metal-containing compounds are also widely used animal feed additives (Cavaco et al., 2011; Argudín et al., 2016). Metals, e.g., copper, zinc, cobalt, chromium, and manganese, are widely used in animal feed for their growth-stimulating and antimicrobial effects; copper and silver are commonly used as disinfectants and preservatives, and mercury, lead, arsenic, and cadmium can be found as contaminants in animal feed (Seiler and Berendonk, 2012; Korish and Attia, 2020). However, the use of heavy metals at high concentrations causes problems due to their toxicity, bioaccumulation, and biomagnification in the food chain (Figure 1; Eisler, 1993). Heavy metal contamination in meat and the prevalence of these pollutants in the environment is a risk for both food safety and human health (Khan et al., 2008; Bamuwamye et al., 2015; Lu et al., 2015). Notably, the use of metals may potentially promote the proliferation of antibiotic resistance through co-selection; the metal and antibiotic resistance are often linked either due to co-location of the resistance genes, a shared resistance mechanism, or co-regulation of resistance pathways (Baker-Austin et al., 2006; Deng et al., 2017; Pal et al., 2017; Yang et al., 2020). The disinfectant resistance genes and heavy metal resistance genes (HMRGs) are commonly located in mobile genetic elements (MGEs) (Frost et al., 2005). The role of metals, antibiotics, and disinfectants in the development and spread of antimicrobial resistance has raised concerns (Bragg et al., 2014; Roosa et al., 2014; Zou et al., 2014; Di Cesare et al., 2016). However, the co-occurrence of heavy metal, antibiotic and disinfectant resistance in *Salmonella* Typhimurium isolates from retail foods, animal manure, and human stool has not been widely investigated. Therefore, we studied the prevalence and associations of resistances of *Salmonella* Typhimurium isolates from pig meat, pig manure, chicken meat, poultry manure, and human stool from Sichuan, China.

## Materials and Methods

### Bacterial Strains

The 300 *Salmonella* Typhimurium strains analyzed in this study were isolated from January 2016 to December 2018 in Sichuan, China, from pig meat (*n* = 182), pig manure (*n* = 23), chicken meat (*n* = 30), poultry manure (*n* = 27), and stool of hospitalized diarrhea patients (*n* = 38). The details of sampling and location of the strains are available in Supplementary Tables 4A,B.

### Isolates and Serotyping

Collected specimens were tested for *Salmonella* using the following protocol. Stool samples from diarrhea patients were enriched in Selenite Brilliant Green broth (SBG, CHROMagar, Paris, France) for 16–22 h at 37°C. For the isolation of *Salmonella* from pig and chicken meat and manure samples, the method described by the United States Department of Agriculture Food Safety and Inspection Service was used (Cui et al., 2006; Guo et al., 2011). Twenty five g portions of meat were used for enrichment and each sample was placed in separate sterile Erlenmeyer flasks with 225 mL buffered peptone water then incubated at 37°C in a water bath with shaking at 120 rpm for 6 h. After pre-enrichment, 1 and 10 mL of pre-enriched solutions were transferred to 100 mL each of the Rappaport-Vassiliadis (RV; Beijing Land Bridge Technology Co., Ltd.) and tetrathionate (TT; Beijing Land Bridge Technology Co., Ltd., Beijing, China) broths, respectively, and incubated at 42°C in a water bath with shaking at 160 rpm for 24 h. One loopful of TT broth and RV broth were streaked onto xylose lysine tergitol agar plates (Beijing Land Bridge Technology Co., Ltd.), and onto agar of xylose lysine deoxycholate (Beijing Land Bridge Technology Co., Ltd.) respectively, and incubated at 37°C for 24 h. Three plausible *Salmonella* colonies from each plate were inoculated onto urea agar slants (Beijing Land Bridge Technology Co., Ltd.) and triple sugar iron (Beijing Land Bridge Technology Co., Ltd.), and incubated at 35°C for 24 h. Further, typical *Salmonella* phenotypes were confirmed by polymerase chain reaction (PCR) as described previously (Cui et al., 2006). A 284 bp PCR product targeting *invA* was amplified using the primers *invA* 139 (5¢-GTGAAATTATCGCCA CGTTCGGGCAA-3¢) and *invA* 141 (5¢-TCATCGCACCGT CAAAGGAACC-3¢) (Deng et al., 2017). Only one isolate from each *Salmonella* positive sample was randomly selected and included in this study (Vo et al., 2006). Confirmed isolates were stored in Tryptone Soya Broth (Hangzhou Microbial Reagent Co., Ltd.) containing 20% glycerol at −80°C until use. Confirmed *Salmonella* isolates were further serotyped according to the Kauffmann-White scheme, by slides using a microtiter agglutination test for O and H antigens, as described in the manufacturer’s instructions (SSI, Copenhagen, Denmark).

### PCR Amplification of Heavy Metal Resistance Genes

The total DNA from *Salmonella* Typhimurium strains was extracted using TIANamp bacteria DNA kit (TIANGEN Biotect (Beijing) Co., Ltd.) according to the manufacturer’s instructions. DNA extractions were stored at −20°C for further analysis. HMRGs *cnrA, nccA, pbrA, pcoA, pcoC, pcoR, chrB, czcB, czcD, arsB, merA*, and *cadD* were amplified using previously published primers (Supplementary Table 1). PCR amplification was carried out in a 25 μL reaction volume, comprising 10 μL of 2 × T5 Super PCR Mix, 2 μL of 5 × Enhancer Buffer, 1 μL of each forward and reverse primer, 2U of Taq-polymerase (Promega, Madison, WI, United States) and 2 μL of template DNA or 2 μL of sterile deionized water as a negative control. The thermal program included initial denaturation at 98°C for 3 min, followed by 34 cycles of denaturation at 98°C for 10 s, annealing at the Tm of primer pair (Supplementary Table 1) for 10 s and extension at 72°C for 20 s, and a final extension at 72°C for 2 min. The success of amplification was verified using electrophoresis in 0.8% agarose gel (GENEI, Bengaluru, India) with a 2,000 bp DNA molecular weight marker as reference (Fermentas, Waltham, MA, United States) and visualized using a gel documentation system (BIO-RAD, Hercules, CA, United States). Appropriate positive controls for amplification were selected from retail meat *Salmonella* Typhimurium isolates. The positive controls were confirmed by sequencing the amplicons (GENEWIZ, Inc., Germantown, MD, United States). All results were confirmed by at least two independent assays.

### Determination of Minimal Inhibitory Concentrations (MICs)

The minimal inhibitory concentrations (MICs) of metal ions, disinfectants, and antibiotics for *Salmonella* Typhimurium were determined using the agar microdilution method as described by the Clinical and Laboratory Standards Institute (CLSI) (Weinstein and Lewis, 2020). Mueller-Hinton agar plates were inoculated with bacteria suspended in 0.85% NaCl to a turbidity equivalent to a 0.5 McFarland using a multipoint inoculator (Oxoid, Lenexa, KS, United States) with approximately 10^4^ CFU per spot. The plates were incubated at 37°C for 18–24 h. The MICs were determined as the lowest concentration of the metal that inhibited the growth of strains completely after 18–24 h of culture at 37°C. All experiments were run in triplicate.

The MICs of copper (CuCl_2_.2H_2_O), chromium (CrCl_3_.6H_2_O), cobalt (CoCl_2_), cadmium (CdCl_2_), zinc (ZnSO_4_), and manganese Mn (MnCl_2_) (Alfa Aesar, Shanghai, China) were determined. Doubling dilutions of the heavy metal stock solutions were incorporated into Mueller-Hinton agar plates with final concentrations ranging from 0.25 to 3200 mg L^–1^. *Escherichia coli* ATCC 10536 and *Salmonella* H9812 were used as the quality control strain in the tests (Zou et al., 2014; Deng et al., 2017; Yang et al., 2020). The MICs of benzalkonium chloride (BC), Trichloroisocyanuric acid (TCCA), (Chengdu Best-Reagent Company, Chengdu, China; > 98% purity), Triclosan (TCS) (J&K Chemical; > 98% purity), were determined at concentration ranges of 0.125-1024 mg L^–1^ for BC and TCCA and 0.03125–1.0 mg L^–1^ for TCS. *Escherichia coli* ATCC 10536 and *Salmonella* H9812 were used as the quality control strain in the tests (Zou et al., 2014; Deng et al., 2017; Yang et al., 2020). In the MIC assays of streptomycin (S), sulfonamides (S3), tetracycline (TET), ampicillin (AMP), nalidixic acid (NA), chloramphenicol (C), sulfamethoxazole (SXT), trimethoprim (TMP), gentamicin (CN), amoxicillin/clavulanic acid (AMC), ciprofloxacin (CIP), ofloxacin (OFX), ceftazidime (CAZ) and cefotaxime (CTX) (Hangzhou Microbial Reagent Co., Ltd., China), the breakpoints for antibiotic resistance and/or susceptibility were determined as recommended by the Clinical and Laboratory Standards Institute (CLSI). *Escherichia coli* ATCC 25922 and 35218 strain were used for quality control (Wang J. et al., 2017).

### Conjugation Experiment

The transfer of HMRGs was determined in a conjugation experiment using mixed broth cultures as previously described (Cai et al., 2008). Isolates with the highest resistance against copper and other metals were chosen as donors. Plasmid-free *Escherichia coli* J53 strain resistant to sodium azide and sensitive to the metals used in this study was selected as a recipient. Donor and recipient strains were grown on trypticase soy agar (TSA) plates overnight, single colonies of donor and recipient were inoculated into 30 mL of Mueller Hinton Broth (MHB) and grown at 37°C for 18 h, after which the recipient and donor strains were mixed at 10:1 (v = v) proportion. Subsequently, 1 mL of the mixture was inoculated onto a sterilized membrane on Mueller Hinton Agar (MHA) and incubated at 37°C for 18 h. The trans-conjugant bacteria were suspended into 3 mL of 0.9% NaCl, and serial dilutions were spread on MHA plates containing 200–400 μg/mL copper and 100 μg/mL sodium azide (Wang et al., 2003; Xu et al., 2007). Plates were incubated at 37°C and inspected at 24 and 48 h. The transfer of heavy metal resistance determinants was determined by amplifying the Cr-Zn-Cd-resistance gene *czcD*, Cu-resistance gene *pcoC*, and Co-Ni-resistance gene *cnrA* using DNA from trans-conjugant bacteria as a template. The MIC of copper (CuCl_2_.2H_2_O) for the trans-conjugants were determined with final concentrations ranging from 100 to 800 μg L^–1^. The conjugation experiment was repeated at least twice.

### Statistical Analysis

Association of the metal resistances and HMRGs with antibiotic and disinfectant resistance in *Salmonella* Typhimurium were determined using the χ^2^-test of independence or Fisher’s exact test was performed to analyze data using SPSS v. 21. *P*-value less than 0.05 was considered statistically significant.

The data about prevalence were analyzed using one-way analysis of variance (ANOVA) and Duncan’s multiple range tests in SAS statistical software (SAS Institute; Cary, NC, United States). Differences were considered statistically significant at *P* ≤ 0.05. The results were visualized using GraphPad prism 8.0.1. (GraphPad Software, San Diego, CA, United States).

## Results

### Metal Resistance

**FIGURE 2:**
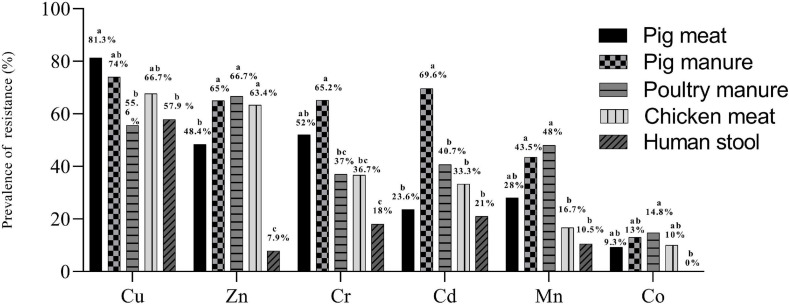
Prevalence of resistance to Cu, Zn, Mn, Cd, Co, and Cr among *Salmonella* Typhimurium isolates from pig meat, pig manure, poultry manure, chicken meat, and human stool. Different lowercase letters indicate statistically significant differences between groups (*P* < 0.05).

Over 98% of the 300 *Salmonella* Typhimurium isolates had MICs of 400–800 mg L^–1^ for Cu, 400–800 mg L^–1^ for Zn, 800–1600 mg L^–1^ for Mn, 25–50 mg L^–1^ for Cd, 200–400 mg L^–1^ for Co and 400–800 mg L^–1^ for Cr (Table 1). Compared to *E. coli* ATCC 10536 and *Salmonella* H9812, 74% (*n* = 222), 47.7% (*n* = 143), 45.7% (*n* = 137), 27.7% (*n* = 83), 29.3% (*n* = 88), and 9% (*n* = 27) of the isolates had higher MIC for Cu, Zn, Cr, Mn, Cd and Co, respectively, and were considered resistant. The prevalence of Cu resistance was higher among the isolates from pig meat than among the human stool and poultry manure isolates (*P* < 0.05) (Figure 2). The prevalence of Zn resistance was highest among the isolates from pig manure, poultry manure, and chicken meat, second highest among the pig meat isolates, and lowest among the human stool isolates (*P* < 0.05).

**TABLE 1:** Incidence of metal resistance among 300 *Salmonella* Typhimurium strains isolated from pig meat, pig manure, poultry manure, chicken meat, and human stool samples.

	**MIC**									**Resistance**
	**12.5**	**25**	**50**	**100**	**200**	**400**	**800**	**1600**	**3200**	**%**
Cu				1	1	76	**222**			74
Zn			4			152	**143**			47.7
Mn			1				216	**83**		27.7
Cd	5	207	**88**							29.3
Co		1		2	270	**27**				9
Cr						163	**137**			45.7

*The strains with MIC higher than that of control strains E. coli ATCC 10536 and Salmonella H9812 were considered resistant. The numbers of resistant strains are in bold.*

The prevalence of Cr resistance was highest among the isolates from pig manure and lowest among the human stool isolates (*P* < 0.05). The prevalence of Cd resistance was higher among the isolates from pig manure than among the other isolates (*P* < 0.05). The prevalence of Mn resistance was highest among the isolates from pig and poultry manure and lowest among the chicken meat and human stool isolates (*P* < 0.05). None of the human stool isolates showed Co resistance and among the other isolates, the prevalence of resistance was on the same level.

In total, 97% (*n* = 291) of the isolates carried at least one heavy metal resistance gene (HMRG). A total of 132 gene combinations were found (Supplementary Table 2). The Cr-Zn-Cd-resistance gene *czcD* was carried by 254 isolates. The Cu-resistance genes *pcoR* and *pcoC* were found in 196 and 179 isolates, respectively. The Co-Ni-resistance gene *cnrA*, Ni-Cr-Cd-resistance gene *nccA*, and Cd-resistance gene *cadD* were carried by 155, 153, and 131 the isolates, respectively. The Hg-resistance gene *merA*, Pb-resistance gene *pbrA*, Cu-resistance gene *pcoA*, and Cr-resistance gene *chrB* were carried by 104, 63, 61, and 18 isolates, respectively. The Cr-Zn-Cd-resistance gene *czcB* and As-resistance gene *arsB* were carried by 17 and 15 isolates (Table 2).

**TABLE 2:** Prevalence of heavy metal resistance genes among 300 *Salmonella* Typhimurium isolates.

**Heavy metal resistance gene**	**Number of isolates (n)**	**Prevalence (%)**
*czcD*	254	84.7
*pcoR*	196	65.3
*pcoC*	179	59.7
*cnrA*	155	51.7
*nccA*	153	51
*cadD*	131	43.7
*merA*	104	34.7
*pbrA*	63	21
*pcoA*	61	20.3
*chrB*	18	6
*czcB*	17	5.7
*arsB*	15	5

**FIGURE 3:**
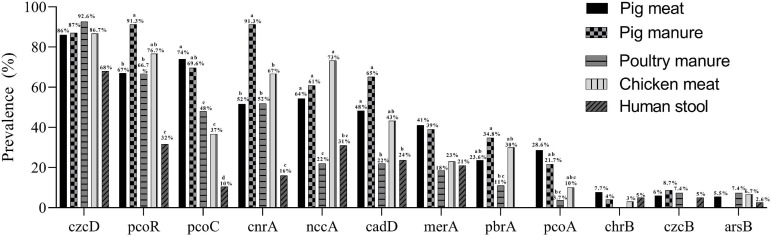
Prevalence of heavy metal resistance genes among *Salmonella* Typhimurium isolates from pig meat, pig manure, poultry manure, chicken meat, and human stool. Different lowercase letters indicate statistically significant differences between groups (*P* < 0.05).

The prevalence of *pcoR* and *cnrA* were highest among the pig manure isolates and lowest among the human stool isolates (*P* < 0.05) (Figure 3). The prevalence of *pcoC* was highest among the pig meat and manure isolates and lowest among the human stool isolates (*P* < 0.05). The prevalence of *nccA* was highest among the chicken meat isolates and lowest among the poultry manure isolates (*P* < 0.05). The prevalence of *cadD* was higher among the pig meat, pig manure, and chicken meat isolates than among the poultry manure and human stool isolates (*P* < 0.05). The prevalence of *pbrA* was higher among the pig manure isolates than among the poultry manure isolates (*P* < 0.05). The prevalence of *pcoA* was higher among the pig meat isolates than among the poultry manure isolates (*P* < 0.05).

### Antibiotic Resistance

A total of 287 *Salmonella* Typhimurium isolates were resistant to at least one antibiotic; all the 23 pig manure isolates, 98.4% of the pig meat isolates (*n* = 179), 97.4% of the human stool isolates (*n* = 37), 86.7% of the chicken meat isolates (*n* = 26) and 81.5% of the poultry manure isolates (*n* = 22). Over 200 isolates were resistant to streptomycin (S), sulfonamides (S3), tetracycline (TET), or ampicillin (AMP), over 120 isolates to nalidixic acid (NA), chloramphenicol (C), sulfamethoxazole (SXT), trimethoprim (TMP) or gentamicin (CN), and less than 55 to amoxicillin/clavulanic acid (AMC), ciprofloxacin (CIP), ofloxacin (OFX), ceftazidime (CAZ) or cefotaxime (CTX) (Table 3).

**TABLE 3:** Prevalence of antibiotic resistance among 300 *Salmonella* Typhimurium isolates.

**Antibiotic resistance**	**Number of isolates**	**Prevalence (%)**
S	237	79
S3	234	78
TET	228	76
AMP	216	72
NA	194	64
C	180	60
SXT	171	57
TMP	165	55
CN	123	41
AMC	54	18
CIP	27	9
OFX	18	6
CAZ	3	1
CTX	3	1

**FIGURE 4:**
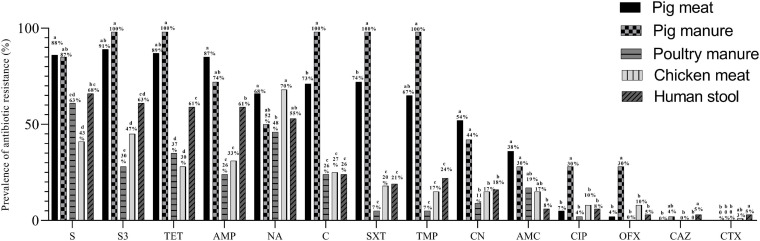
Prevalence of antibiotic resistance among *Salmonella* Typhimurium isolates from pig meat, pig manure, poultry manure, chicken meat, and human stool. Different lowercase letters indicate statistically significant differences between groups (*P* < 0.05).

The prevalence of resistance to S and AMP was higher among the pig meat isolates than among the poultry manure, chicken meat, and human stool isolates (*P* < 0.05) (Figure 4). The prevalence of resistance to S3, TET, C, SXT, TMP, and CN was highest among the pig meat and manure isolates (*P* < 0.05). The prevalence of resistance to CIP and OFX was highest among the pig manure isolates (*P* < 0.05). The prevalence of resistance to AMC was higher among the pig meat and manure isolates than among the human stool isolates (*P* < 0.05). The prevalence of resistance to NA was higher among the pig and chicken meat isolates than among the poultry manure isolates (*P* < 0.05). None of the pig and chicken meat and poultry manure isolates were resistant to CAZ, and none of the pig meat and pig and poultry manure isolates were resistant to CTX.

A total of 77 resistance profiles were observed among the isolates. A multiple resistance profile TET-AMP-SXT-C-NA-TMP-CN-S3-S was carried by 36 isolates, TET-AMP-SXT-C-TMP-S3-S by 25 isolates, TET-AMC-AMP-SXT-C-NA-TMP-CN-S3-S by 23 isolates, and TET-AMP-S3-S by 16 isolates (Supplementary Table 3).

A total of 238 isolates were multidrug-resistant (MDR) with resistance to at least three classes of antibiotics. The prevalence of MDR was highest among pig manure isolates (100%, *n* = 23) followed by pig meat isolates (92.3%, *n* = 168), human stool isolates (68.4%, *n* = 26), poultry manure isolates (40.7%, *n* = 11) and chicken meat isolates (36.6%, *n* = 11).

### Disinfectant Resistance

**FIGURE 5:**
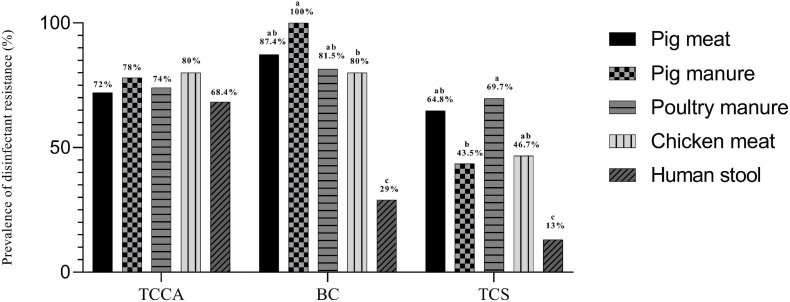
Prevalence of disinfectant resistance among *Salmonella* Typhimurium isolates from pig meat, pig manure, poultry manure, chicken meat, and human stool. Different lowercase letters indicate statistically significant differences between groups (*P* < 0.05).

The MICs of *E. coli* ATCC 10536 and *Salmonella* H9812 for benzalkonium chloride (BC), trichloroisocyanuric acid (TCCA), and triclosan (TCS) were 8, 256, and 0.0625, mg L^–1^, respectively. A total of 238, 218, and 164 of the *Salmonella* Typhimurium isolates had higher MICs for BC, TCCA, and TCS, respectively, than *E. coli* ATCC 10536 and *Salmonella* H9812 (Table 4) and were considered resistant. The prevalence of BC and TCS resistance was lowest among the human stool isolates (*P* < 0.05) (Figure 5). The prevalence of BC resistance was higher among the pig manure isolates than among the chicken meat isolates (*P* < 0.05), whereas the prevalence of TCS resistance was lower among the pig manure isolates than among the poultry manure isolates (*P* < 0.05).

**TABLE 4:** Incidence of disinfectants among 300 *Salmonella* Typhimurium strains isolated from pig meat, pig manure, poultry manure, chicken meat, and human stool samples.

	**MIC**										**Resistance**
	**2**	**4**	**8**	**16**	**32**	**64**	**128**	**256**	**512**	**1024**	**%**
BC	17	3	42	**181**	**18**	**39**					79.3
TCCA					33		4	45	**149**	**69**	72.7

	**MIC**										**Resistance**
	**0.0313**		**0.0625**		**0.125**		**0.25**		**0.5**	**1**	

Triclosan (TCS)	112		24		**46**		**77**		**39**	**2**	55

*The strains with MIC higher than that of control strain E. coli ATCC 10536 and Salmonella H9812 were considered resistant. The numbers of resistant strains are in bold.*

### Association of Metal Resistance and Heavy Metal Resistance Genes With Antibiotic and Disinfectant Resistance

All the six metal resistances were associated with resistance to at least one antibiotic (Table 5 and Supplementary Table 5A). Cr-resistance was associated with resistance to eight antibiotics and to two disinfectants, Cu-resistance was associated with resistance to eight antibiotics, and Zn-resistance was associated with resistance to six antibiotics and to two disinfectants.

**TABLE 5:** The association of metal resistances with antibiotic and disinfectant resistance in *Salmonella* Typhimurium.

**Metal**	**Antibiotic/Disinfectant**	***P*-value**
	**Antibiotic**	
Cd	CIP	<0.00001
	OFX	<0.00001
	CAZ	0.0003
Co	AMP	0.0229
Cr	AMP	0.0019
	SXT	<0.00001
	CIP	<0.05
	C	0.0329
	OFX	0.0002
	TMP	<0.05
	CN	0.0028
	S3	0.0167
Cu	TET	<0.00001
	AMP	0.0001
	SXT	<0.00001
	C	0.0007
	TMP	<0.00001
	CN	0.0002
	S3	0.0022
	S	<0.00001
Mn	AMP	0.044
	SXT	0.004
	TMP	0.0279
	CN	0.0013
Zn	SXT	0.0036
	CIP	0.0019
	C	0.0249
	OFX	0.0007
	TMP	0.0037
	CN	0.0062
	**Disinfectant**	
Cd	BC	0.0397
Co	BC	0.0227
Cr	BC	0.0303
	TCCA	<0.00001
Mn	BC	0.0012
Zn	BC	<0.00001
	TCCA	<0.05

All the 12 HMRGs were associated with resistance to at least two antibiotics (Table 6 and Supplementary Table 5B). The Hg-resistance gene *merA* was associated with resistance to 12 antibiotics and to three disinfectants, the Cu-resistance gene *pcoA* with resistance to ten antibiotics and to two disinfectants, the Pb-resistance gene *pbrA* with resistance to seven antibiotics and to two disinfectants, and the Ni-Cr-Cd-resistance gene *nccA* and the Cu-resistance gene *pcoC* with resistance to seven antibiotics and to one disinfectant (*P* < 0.05).

**TABLE 6:** The association of HMRGs with antibiotic and disinfectant resistance in *Salmonella* Typhimurium.

**HMRG**	**Antibiotic/Disinfectant**	***P*-value**
	**Antibiotic**	
cadD	AMP	0.0064
	SXT	<0.00001
	S3	0.0106
	TMP	0.0002
chrB	AMP	0.0293
	TMP	0.0135
cnrA	SXT	0.0003
	CIP	0.0075
	OFX	0.0101
	TMP	0.0001
merA	TET	<0.00001
	AMC	0.0044
	AMP	<0.00001
	SXT	0.0033
	CIP	0.0003
	C	0.0063
	OFX	0.0001
	NA	0.0079
	TMP	0.0286
	CN	<0.00001
	S3	<0.00001
	S	0.011
nccA	TET	0.0313
	AMP	0.0144
	SXT	0.0072
	OFX	0.0356
	NA	0.0119
	TMP	0.0106
	CN	0.001
pbrA	SXT	0.0009
	CIP	0.0005
	C	0.044
	OFX	0.0001
	NA	0.0012
	TMP	0.0069
	CN	0.0005
pcoA	TET	0.0002
	AMC	0.0083
	AMP	0.000114
	SXT	<0.00001
	CIP	0.0375
	C	0.0081
	NA	0.0369
	TMP	0.0003
	CN	0.0002
	S3	0.0002
pcoC	TET	0.000012
	AMP	<0.00001
	SXT	<0.00001
	C	<0.00001
	TMP	<0.00001
	CN	0.000025
	S3	0.000024
pcoR	SXT	0.0098
	CIP	0.0002
	OFX	0.0027
	TMP	0.0104
cnrA	BC	0.0066
merA	BC	0.0349
	TCS	0.000027
	TCCA	0.0007
nccA	BC	0.0016
pbrA	BC	0.0013
	TCCA	<0.00001
pcoA	BC	0.0072
	TCCA	0.000045
pcoC	BC	0.0004
pcoR	BC	0.0005
	TCCA	0.0214

### Transfer of Heavy Metal Resistance Genes

**FIGURE 6:**
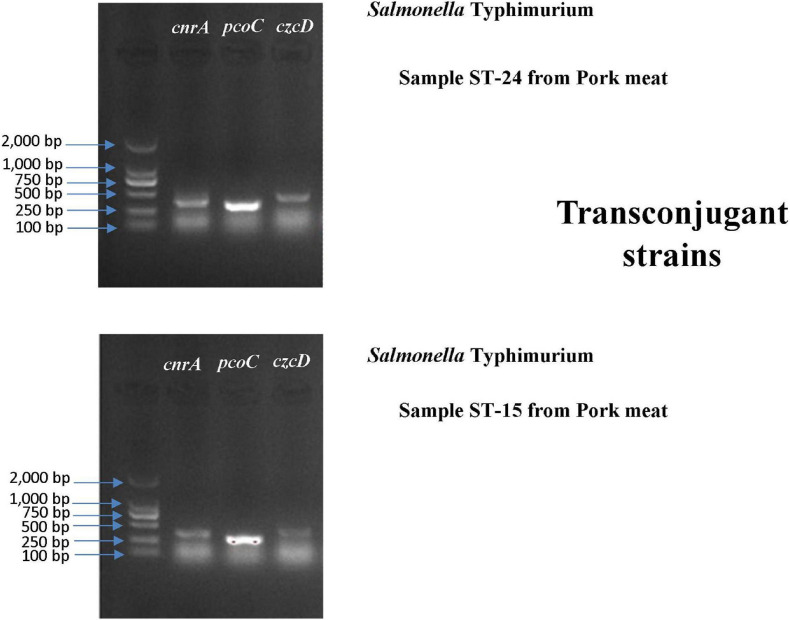
Gel electrophoresis verification of the transfer of *cnrA, pcoC* and *czcD* from *Salmonella* Typhimurium isolates S15 and S24 to *Escherichia coli* J53.

A conjugation experiment was carried out to determine the transferability of HMRGs. The genes *cnrA, pcoC*, and *czcD* were successfully transferred from *Salmonella* Typhimurium isolates S15 and S24 to *Escherichia coli* J53 (Figure 6). The transfer rate was 1 × 10^–3^ per donor. The MIC of Cu was 100 μg L^–1^ for the *Escherichia coli* J53, 800 μg L^–1^ for the isolates S15 and S24, 200 μg L^–1^ for the J53 with genes transferred from S15 and 300 μg L^–1^ for J53 with genes transferred from S24 (Table 7).

**TABLE 7:** Transconjugation of metal resistance genes from *Salmonella* Typhimurium to *Escherichia coli* J53.

**Strain number**		**Genes**	**MIC of Cu (μg L^–1^)**				
			**100**	**200**	**300**	**400**	**800**
Recipient	*Escherichia coli*						
	J53		100				
Donor	*Salmonella* Typhimurium						
	ST-24	*cnrA, pcoC, czcD*					800
	ST-15	*CnrA, pcoC, czcD*					800
Transconjugant	*Escherichia coli*						
	J53-ST-24	*cnrA, pcoC, czcD*			300		
	J53-ST-15	*cnrA, pcoC, czcD*		200			

## Discussion

The increasing resistance of *Salmonella* strains to antimicrobial agents has become a major public health concern worldwide. However, little information is available on the resistance and co-resistance to heavy metals, disinfectants, and antibiotics among *Salmonella* Typhimurium from retail meat and manure. Thus, we studied the prevalence and associations of resistances of *Salmonella* Typhimurium isolate from pig meat, pig manure, chicken meat, poultry manure, and human stool from Sichuan, China.

In our study, 74% of the 300 *Salmonella* Typhimurium isolates were considered resistant to Cu, almost 50% to Zn and Cr, over 25% to Mn and Cd, and almost 10% to Co. Approximately similar prevalence of Cu resistant isolates has been detected among *Salmonella enterica* isolates from meat and meat-based products (Figueiredo et al., 2019). The high prevalences of Cu and Zn resistant isolates may have been due to selection by heavy metal micronutrients in the feed; the use of heavy metal micronutrients resulted in high concentrations of Cu and Zn in pig feces in the U.S. (Medardus et al., 2014). Alarmingly, the resistant strains in the feces may contaminate the meat, as suggested by the high Cu resistance prevalence among the pig and chicken meat isolates. The high prevalence of resistant manure isolates suggested that the resistance may spread further by the use of manure as a soil amendment in agriculture. Further studies are needed to ascertain whether the high prevalence of Cu resistance among human stool isolates had resulted from meat products.

In line with the prevalence of metal resistances, most of the isolates carried at least one heavy metal resistance gene (HMRG), and the prevalences of the Cu-resistance genes *pcoR* and *pcoC* and Cu resistance were at the same level. HMRGs are found in a wide variety of bacteria from various environments (Li L.-G. et al., 2017; Pal et al., 2017). The *czcD* gene is involved in the regulation of an efflux system that mediates the resistance to metal ions (Anton et al., 1999). Almost 70% of the *Salmonella* Typhimurium isolates from pig feed and feces carried *czcD* (Medardus et al., 2014). In our study, the prevalence of the Cr-Zn-Cd-resistance gene *czcD* was 85%, higher than that of resistances to those metals. Possibly the gene was not effectively expressed under the test conditions. However, ascertaining this necessitates further analyses.

The metal resistant isolates are often also antibiotic and disinfectant resistant; the resistance genes may be co-located, e.g., on a plasmid, or the resistance mechanism, e.g., an efflux pump, may provide resistance against both metals, and antibiotics (Deng et al., 2017; Pal et al., 2017). In our study, most of the *Salmonella* Typhimurium isolates were resistant to at least one antibiotic and almost 80% were multidrug-resistant. Similarly, among *Salmonella* isolates from retail food of animal origin in Romania and China, over 90% were resistant to at least one antibiotic, and from 43 to over 80% were multidrug-resistant (Mihaiu et al., 2014; Deng et al., 2017). The prevalence of resistance to streptomycin, sulfonamides, tetracycline, and ampicillin were all over 70% among our isolates. In agreement with Deng et al. (2017), pigs were a major source of antibiotic-resistant isolates. The prevalence of resistance to six antibiotics was higher among the pig meat and manure isolates than among other isolates, and that of streptomycin and ampicillin were highest among the pig meat isolates and that of ciprofloxacin and ofloxacin among the pig manure isolates. In our study, from 55 to 79% of the isolates were considered resistant to disinfectants triclosan (TCS), trichloroisocyanuric acid (TCCA), or benzalkonium chloride (BC). The MICs for BC resistance were lower than those of the meat *Salmonella* isolates, among which almost 60% had a MIC of 128 mg L^–1^ for BC (Deng et al., 2017). For three of the disinfectants, the prevalence of resistance was lowest among the human stool isolates, suggesting that the resistance had not been passed on in the food chain.

Due to the genes and mechanisms shared between metal, antibiotic, and disinfectant resistance, the development of resistance against metals may be associated with the development of antibiotic and disinfectant resistance (Deng et al., 2017; Pal et al., 2017). Similar to previous studies (Deng et al., 2017; Di Cesare et al., 2016; Yang et al., 2018), in our study, the metal resistances and HMRGs were associated with resistance to antibiotics and disinfectants. Especially, Cu-resistance genes were associated with resistance to several antibiotics and disinfectants. Thus, the excessive use of metals and disinfectants as feed additives and in animal care may have the potential to promote antibiotic resistance through co-selection. Alarmingly, this co-selection can maintain and promote antibiotic resistance even in the absence of antibiotics, and e.g., Cu may co-select for resistance to last-resort antibiotics such as colistin (Pal et al., 2017).

Knowing the genetic linkage of resistance genes and their association with MGEs is critical to fully understand the risks of horizontal transfer of resistance genes between bacteria (Martínez et al., 2015; Pal et al., 2015). The operons encoding resistance against different metals have been confirmed to be located on the same plasmid (Fang et al., 2016). In our study, the transfer of the Cr-Zn-Cd-resistance gene *czcD*, Cu-resistance gene *pcoC*, and Co-Ni-resistance gene *cnrA* into *Escherichia coli* and the increased Cu-resistance of the transconjugants implied that the resistance genes were located on conjugative plasmids and may be expressed in a receiving strain. Similarly, Hasman and Aarestrup (2002) and Amachawadi et al. (2013) reported that the copper resistance gene *tcrB* is horizontally transferable and linked to macrolide and glycopeptide resistance. Thus, the metal resistance genes may spread between bacteria and, due to the connection between metal and antibiotic resistance, further increase the prevalence of antibiotic resistance in the environment.

The study had the few limitations. Firstly, *Salmonella* Typhimurium, as a control strain, was not available and therefore we used standard control strains like *Escherichia coli* ATCC 25922, *Escherichia coli* ATCC 10536 and *Salmonella* H9812 as some previous studies who did the same in a scenario where the control strain was not available due to the reason such as faced by us.

Secondly, understanding differences in metal tolerances among pig feces isolates is certainly worthwhile and could have been done. However, group-wise comparison has previously been performed by a few researchers before with some limitations. In the current study, we made group-wise comparison and tried answering some of the limitations that were found in those previous studies. Since our group is constantly working on this particular aspect, future study will certainly be conducted in the subject manner as proposed.

## Conclusion

We found a co-occurrence of heavy metal, antibiotic and disinfectant resistance in *Salmonella* Typhimurium isolates originating from retail foods, animal manure, and human stool. Such an increased prevalence of metal resistance and its corresponding genes among *Salmonella* Typhimurium isolates has not been reported previously and therefore provides a baseline study to further investigate the subject matter. Further, a prevalence of resistance and its genes indicates that meat and manure could be potential sources of human exposure to multiple strains of resistant *Salmonella* and other food-borne diseases. An excessive as well as an irrational use of metals and disinfectants either as feed additives or in an animal care setting may promote antibiotic resistance through co-selection and the transfer of the resistance genes through MGEs.

## Data Availability Statement

The original contributions presented in the study are included in the article/Supplementary Material, further inquiries can be directed to the corresponding author/s.

## Author Contributions

GM and KZ wrote the manuscript under the supervision of LZ. GM, KZ, XH, SC, SL, AM, LH, YY, XY, XA, AL, and XX performed sample collection and research work. GM and KZ performed data analysis. GM, KZ, LZ, MS, PP, and AM revised the manuscript. LZ was correspond to the author furthest up on the author list. All authors approved the manuscript’s final version.

## Conflict of Interest

The authors declare that the research was conducted in the absence of any commercial or financial relationships that could be construed as a potential conflict of interest.

## Publisher’s Note

All claims expressed in this article are solely those of the authors and do not necessarily represent those of their affiliated organizations, or those of the publisher, the editors and the reviewers. Any product that may be evaluated in this article, or claim that may be made by its manufacturer, is not guaranteed or endorsed by the publisher.

## References

[B1] Allen H. K. Levine U. Y. Looft T. Bandrick M. Casey T. A. (2013). Treatment, promotion, commotion: antibiotic alternatives in food-producing animals. *Trends Microbiol.* 21 114–119. 10.1016/j.tim.2012.11.001 23473629

[B2] Amachawadi R. G. Scott H. M. Alvarado C. A. Mainini T. R. Vinasco J. Drouillard J. S. (2013). Occurrence of the transferable copper resistance Gene tcrB among fecal enterococci of U.S. feedlot cattle fed copper-supplemented diets. *Appl. Environ. Microbiol.* 79 4369–4375. 10.1128/aem.00503-13 23666328 PMC3697488

[B3] Anton A. Große C. Reißmann J. Pribyl T. Nies D. H. (1999). CzcD is a heavy metal ion transporter involved in regulation of heavy metal resistance in Ralstonia sp. strain CH34. *J. Bacteriol.* 181 6876–6881. 10.1128/jb.181.22.6876-6881.1999 10559151 PMC94160

[B4] Antunes P. Mourão J. Campos J. Peixe L. (2016). Salmonellosis: the role of poultry meat. *Clin. Microbiol. Infect.* 22 110–121. 10.1016/j.cmi.2015.12.004 26708671

[B5] Argudín M. A. Lauzat B. Kraushaar B. Alba P. Agerso Y. Cavaco L. (2016). Heavy metal and disinfectant resistance genes among livestock-associated methicillin-resistant *Staphylococcus aureus* isolates. *Vet. Microbiol.* 191 88–95. 10.1016/j.vetmic.2016.06.004 27374912

[B6] Baker-Austin C. Wright M. S. Stepanauskas R. McArthur J. V. (2006). Co-selection of antibiotic and metal resistance. *Trends Microbiol.* 14 176–182. 10.1016/j.tim.2006.02.006 16537105

[B7] Bamuwamye M. Ogwok P. Tumuhairwe V. (2015). Cancer and non-cancer risks associated with heavy metal exposures from street foods: evaluation of roasted meats in an urban setting. *J. Environ. Pollut. Hum. Health* 3 24–30.

[B8] Barr W. Smith A. (2014). Acute diarrhea in adults. *Am. Fam. Physician* 89 180–189. 24506120

[B9] Barton Behravesh C. Jones T. F. Vugia D. J. Long C. Marcus R. Smith K. (2011). Deaths associated with bacterial pathogens transmitted commonly through food: foodborne diseases active surveillance network (FoodNet), 1996–2005. *J. Infect. Dis.* 204 263–267. 10.1093/infdis/jir263 21673037

[B10] Bragg R. Jansen A. Coetzee M. van der Westhuizen W. Boucher C. (2014). “Bacterial resistance to quaternary ammonium compounds (QAC) disinfectants,” in *Infectious Diseases and Nanomedicine II Advances in Experimental Medicine and Biology*, eds Adhikari R. Thapa S. (New Delhi: Springer), 1–13. 10.1007/978-81-322-1774-9_1 24595606

[B11] Burch D. (2006). Anticipated Effects of the Withdrawal of Antibiotic Growth Promoters (AGPs) From Pigs in the European Union on 1st January 2006. Available online at: http://www.octagon-services.co.uk/articles/withdrawalAGP.htm

[B12] Cai J. C. Zhou H. W. Zhang R. Chen G.-X. (2008). Emergence of *Serratia marcescens*, *Klebsiella pneumoniae*, and *Escherichia coli* Isolates possessing the plasmid-mediated carbapenem-hydrolyzing beta-lactamase KPC-2 in intensive care units of a Chinese hospital. *Antimicrob. Agents Chemother.* 52 2014–2018. 10.1128/aac.01539-07 18332176 PMC2415814

[B13] Cavaco L. M. Hasman H. Aarestrup F. M. Wagenaar J. A. Graveland H. Veldman K. (2011). Zinc resistance of Staphylococcus aureus of animal origin is strongly associated with methicillin resistance. *Vet. Microbiol.* 150 344–348. 21411247 10.1016/j.vetmic.2011.02.014

[B14] Ceyssens P.-J. Mattheus W. Vanhoof R. Bertrand S. (2015). Trends in serotype distribution and antimicrobial susceptibility in *Salmonella enterica* isolates from humans in Belgium, 2009 to 2013. *Antimicrob. Agents Chemother.* 59 544–552. 10.1128/aac.04203-14 25385108 PMC4291397

[B15] Chousalkar K. Gast R. Martelli F. Pande V. (2018). Review of egg-related salmonellosis and reduction strategies in United States, Australia, United Kingdom and New Zealand. *Crit. Rev. Microbiol.* 44 290–303. 10.1080/1040841x.2017.1368998 28903617

[B16] CIIN (2018a). *Analysis of the Development Status and Development Trend of China’s pig Breeding Industry in 2018 [图]_中国工业信息网* Available Online at: https://www.chyxx.com/industry/201801/603323.html (accessed June 3, 2021)

[B17] CIIN (2018b). *Analysis of the Scale of Pig Breeding in China and the Development Potential of Pig Production Areas in Henan Province in 2017 (Figure)_中国工业信息网* Available Online at: https://www.chyxx.com/industry/201808/667438.html (accessed June 3, 2021)

[B18] Cui S. Zheng J. Meng J. (2006). An improved method for rapid isolation of Salmonella against Proteus in chicken carcasses. *J. Food Saf.* 26 49–61. 10.1111/j.1745-4565.2005.00032.x

[B19] Demirbilek S. K. (2017). Salmonellosis in animals. *Salmonella Re-Emerg. Pathog.* 10.5772/intechopen.72192 Available online at: https://www.intechopen.com/books/salmonella-a-re-emerging-pathogen/salmonellosis-in-animals

[B20] Deng W. Quan Y. Yang S. Guo L. Zhang X. Liu S. (2017). Antibiotic resistance in salmonella from retail foods of animal origin and its association with disinfectant and heavy metal resistance. *Microb. Drug Resist.* 24 782–791. 10.1089/mdr.2017.0127 29039715

[B21] Dewey-Mattia D. Manikonda K. Chen J. Kisselburgh H. Pilewski C. Sundararaman P. (2018). *Surveillance for Foodborne Disease Outbreaks–United States, 2016: Annual Report.* U.S. Centers for Disease Control and Prevention (CDC), Atlanta, Georgia.

[B22] Di Cesare A. Eckert E. M. D’Urso S. Bertoni R. Gillan D. C. Wattiez R. (2016). Co-occurrence of integrase 1, antibiotic and heavy metal resistance genes in municipal wastewater treatment plants. *Water Res.* 94 208–214. 10.1016/j.watres.2016.02.049 26945964

[B23] EFSA and ECDC (2018). The European Union summary report on trends and sources of zoonoses, zoonotic agents and food-borne outbreaks in 2017. *EFSa J.* 16:e05500. 32625785 10.2903/j.efsa.2018.5500 PMC7009540

[B24] Eisler R. (1993). *Zinc Hazards to Fish, Wildlife, and Invertebrates: a Synoptic Review.* Washington, DC: US Department of the Interior.

[B25] Fang L. Li X. Li L. Li S. Liao X. Sun J. (2016). Co-spread of metal and antibiotic resistance within ST3-IncHI2 plasmids from E. coli isolates of food-producing animals. *Sci. Rep.* 6:25312. 27143648 10.1038/srep25312 PMC4855149

[B26] Figueiredo R. Card R. M. Nunez-Garcia J. Mendonça N. da Silva G. J. Anjum M. F. (2019). Multidrug-resistant *Salmonella enterica* isolated from food animal and foodstuff may also be less susceptible to heavy metals. *Foodborne Pathog. Dis.* 16 166–172. 10.1089/fpd.2017.2418 30480469

[B27] Frost L. S. Leplae R. Summers A. O. Toussaint A. (2005). Mobile genetic elements: the agents of open source evolution. *Nat. Rev. Microbiol.* 3 722–732. 10.1038/nrmicro1235 16138100

[B28] Guo C. Hoekstra R. M. Schroeder C. M. Pires S. M. Ong K. L. Hartnett E. (2011). Application of Bayesian techniques to model the burden of human salmonellosis attributable to U.S. food commodities at the point of processing: adaptation of a Danish model. *Foodborne Pathog. Dis.* 8 509–516. 10.1089/fpd.2010.0714 21235394 PMC3123837

[B29] Hasman H. Aarestrup F. M. (2002). tcrB, a gene conferring transferable copper resistance in *Enterococcus faecium*: occurrence, transferability, and linkage to macrolide and glycopeptide resistance. *Antimicrob. Agents Chemother.* 46 1410–1416. 10.1128/aac.46.5.1410-1416.2002 11959576 PMC127162

[B30] He Y. Yang X. Xia J. Zhao L. Yang Y. (2016). Consumption of meat and dairy products in China: a review. *Proc. Nutr. Soc.* 75 385–391. 10.1017/s0029665116000641 27334652

[B31] Heredia N. García S. (2018). Animals as sources of food-borne pathogens: a review. *Anim. Nutr.* 4 250–255. 10.1016/j.aninu.2018.04.006 30175252 PMC6116329

[B32] Hu Y. J. Cowling B. J. (2020). Reducing antibiotic use in livestock, China. *Bull. World Health Organ.* 98 360–361. 10.2471/blt.19.243501 32514201 PMC7265937

[B33] Hugas M. Beloeil P. A. (2014). Controlling Salmonella along the food chain in the European Union - progress over the last ten years. *Eurosurveillance* 19:20804. 24852953 10.2807/1560-7917.es2014.19.19.20804

[B34] Khan S. Cao Q. Zheng Y. M. Huang Y. Z. Zhu Y. G. (2008). Health risks of heavy metals in contaminated soils and food crops irrigated with wastewater in Beijing, China. *Environ. Pollut.* 152 686–692. 10.1016/j.envpol.2007.06.056 17720286

[B35] Korish M. A. Attia Y. A. (2020). Evaluation of heavy metal content in feed, litter, meat, meat products, liver, and table eggs of chickens. *Animals* 10:727. 10.3390/ani10040727 32331361 PMC7222721

[B36] Kumar D. Pornsukarom S. Thakur S. (2019). “Antibiotic usage in poultry production and antimicrobial-resistant Salmonella in poultry,” in *Food Safety in Poultry Meat Production Food Microbiology and Food Safety*, eds Venkitanarayanan K. Thakur S. Ricke S. C. (Cham: Springer), 47–66. 10.1007/978-3-030-05011-5_3

[B37] Kumar K. Gupta S. C. Baidoo S. K. Chander Y. Rosen C. J. (2005). Antibiotic uptake by plants from soil fertilized with animal manure. *J. Environ. Qual.* 34 2082–2085. 10.2134/jeq2005.0026 16221828

[B38] Li L.-G. Xia Y. Zhang T. (2017). Co-occurrence of antibiotic and metal resistance genes revealed in complete genome collection. *ISME J.* 11 651–662. 10.1038/ismej.2016.155 27959344 PMC5322307

[B39] Li W. Li Y. Liu Y. Shi X. Jiang M. Lin Y. (2017). Clonal expansion of biofilm-forming *Salmonella typhimurium* ST34 with multidrug-resistance phenotype in the southern coastal region of China. *Front. Microbiol.* 8:2090. 29163392 10.3389/fmicb.2017.02090 PMC5674920

[B40] Liang Z. Ke B. Deng X. Liang J. Ran L. Lu L. (2015). Serotypes, seasonal trends, and antibiotic resistance of non-typhoidal Salmonella from human patients in Guangdong Province, China, 2009–2012. *BMC Infect. Dis.* 15:53. 25881319 10.1186/s12879-015-0784-4 PMC4343067

[B41] Lu Y. Song S. Wang R. Liu Z. Meng J. Sweetman A. J. (2015). Impacts of soil and water pollution on food safety and health risks in China. *Environ. Int.* 77 5–15. 10.1016/j.envint.2014.12.010 25603422

[B42] Luo Q. Li S. Liu S. Tan H. (2017). Foodborne illness outbreaks in China, 2000–2014. *Int. J. Clin. Exp. Med.* 10 5821–5831.

[B43] Majowicz S. E. Musto J. Scallan E. Angulo F. J. Kirk M. O’Brien S. J. (2010). The global burden of nontyphoidal Salmonella gastroenteritis. *Clin. Infect. Dis.* 50 882–889. 20158401 10.1086/650733

[B44] Martínez J. L. Coque T. M. Baquero F. (2015). What is a resistance gene? ranking risk in resistomes. *Nat. Rev. Microbiol.* 13 116–123. 10.1038/nrmicro3399 25534811

[B45] Medardus J. J. Molla B. Z. Nicol M. Morrow W. M. Rajala-Schultz P. J. Kazwala R. (2014). In-feed use of heavy metal micronutrients in US swine production systems and its role in persistence of multidrug-resistant salmonellae. *Appl. Environ. Microbiol.* 80 2317–2325. 10.1128/aem.04283-13 24487542 PMC3993138

[B46] Mihaiu L. Lapusan A. Tanasuica R. Sobolu R. Mihaiu R. Oniga O. (2014). First study of Salmonella in meat in Romania. *J. Infect. Dev. Ctries.* 8 050–058. 10.3855/jidc.3715 24423712

[B47] Pal C. Asiani K. Arya S. Rensing C. Stekel D. J. Larsson D. J. (2017). Metal resistance and its association with antibiotic resistance. *Adv. Microb. Physiol.* 70 261–313. 10.1016/bs.ampbs.2017.02.001 28528649

[B48] Pal C. Bengtsson-Palme J. Kristiansson E. Larsson D. J. (2015). Co-occurrence of resistance genes to antibiotics, biocides and metals reveals novel insights into their co-selection potential. *BMC Genomics* 16:964. 26576951 10.1186/s12864-015-2153-5 PMC4650350

[B49] Pan H. Li X. Fang W. Yue M. (2018). Analysis of major human and foodborne pathogens and their resistance to antimicrobials in the USA in the past two decades: implications for surveillance and control of antimicrobial resistance in China. *J. Zhejiang Univ. Agric. Life Sci.* 44 237–246.

[B50] Pan H. Zhou X. Chai W. Paudyal N. Li S. Zhou X. (2019). Diversified sources for human infections by *Salmonella enterica* serovar newport. *Transbound. Emerg. Dis.* 66 1044–1048. 10.1111/tbed.13099 30548172 PMC6634944

[B51] Ran L. Wu S. Gao Y. Zhang X. Feng Z. Wang Z. (2011). Laboratory-based surveillance of nontyphoidal Salmonella infections in China. *Foodborne Pathog. Dis.* 8 921–927. 21492026 10.1089/fpd.2010.0827

[B52] Roosa S. Wattiez R. Prygiel E. Lesven L. Billon G. Gillan D. C. (2014). Bacterial metal resistance genes and metal bioavailability in contaminated sediments. *Environ. Pollut.* 189 143–151. 10.1016/j.envpol.2014.02.031 24662000

[B53] Seiler C. Berendonk T. U. (2012). Heavy metal driven co-selection of antibiotic resistance in soil and water bodies impacted by agriculture and aquaculture. *Front. Microbiol.* 3:399. 23248620 10.3389/fmicb.2012.00399 PMC3522115

[B54] Shen H. Chen H. Ou Y. Huang T. Chen S. Zhou L. (2020). Prevalence, serotypes, and antimicrobial resistance of Salmonella isolates from patients with diarrhea in Shenzhen, China. *BMC Microbiol.* 20:197. 32631309 10.1186/s12866-020-01886-5 PMC7339465

[B55] Simpson K. M. Hill-Cawthorne G. A. Ward M. P. Mor S. M. (2018). Diversity of Salmonella serotypes from humans, food, domestic animals and wildlife in New South Wales. Australia. *BMC Infect. Dis.* 18:623. 30518339 10.1186/s12879-018-3563-1 PMC6280480

[B56] Sodagari H. R. Wang P. Robertson I. Habib I. Sahibzada S. (2020). Non-Typhoidal Salmonella at the human-food-of-animal-origin interface in Australia. *Animals* 10:1192. 10.3390/ani10071192 32674371 PMC7401514

[B57] Takaya A. Yamamoto T. Tokoyoda K. (2020). Humoral immunity vs. Salmonella. *Front. Immunol.* 10:3155. 32038650 10.3389/fimmu.2019.03155 PMC6985548

[B58] Tam C. C. Rodrigues L. C. Viviani L. Dodds J. P. Evans M. R. Hunter P. R. (2012). Longitudinal study of infectious intestinal disease in the UK (IID2 study): incidence in the community and presenting to general practice. *Gut* 61 69–77. 10.1136/gut.2011.238386 21708822 PMC3230829

[B59] Vo A. T. Van Duijkeren E. Fluit A. C. Heck M. E. Verbruggen A. Maas H. M. (2006). Distribution of *Salmonella enterica* serovars from humans, livestock and meat in Vietnam and the dominance of Salmonella Typhimurium phage type 90. *Vet. Microbiol.* 113 153–158. 10.1016/j.vetmic.2005.10.034 16337754

[B60] Wang J. Li Y. Xu X. Liang B. Wu F. Yang X. (2017). Antimicrobial resistance of *Salmonella enterica* serovar Typhimurium in Shanghai, China. *Front. Microbiol.* 8:510. 28400764 10.3389/fmicb.2017.00510 PMC5368216

[B61] Wang M. Tran J. H. Jacoby G. A. Zhang Y. Wang F. Hooper D. C. (2003). Plasmid-mediated quinolone resistance in clinical isolates of *Escherichia coli* from Shanghai, China. *Antimicrob. Agents Chemother.* 47 2242–2248. 10.1128/aac.47.7.2242-2248.2003 12821475 PMC161834

[B62] Wang Y. Cao C. Alali W. Q. Cui S. Li F. Zhu J. (2017). Distribution and antimicrobial susceptibility of foodborne Salmonella serovars in eight provinces in China from 2007 to 2012 (except 2009). *Foodborne Pathog. Dis.* 14 393–399. 10.1089/fpd.2016.2237 28375673

[B63] Weinstein M. P. Lewis J. S. (2020). The clinical and laboratory standards institute subcommittee on antimicrobial susceptibility testing: background, organization, functions, and processes. *J. Clin. Microbiol.* 58:e1864-19. 10.1128/JCM.01864-19 PMC7041576 31915289

[B64] WHO (2020). *Food Safety.* Geneva: WHO.

[B65] Xu X. Wu S. Ye X. Liu Y. Shi W. Zhang Y. (2007). Prevalence and expression of the plasmid-mediated quinolone resistance determinant qnrA1. *Antimicrob. Agents Chemother.* 51 4105–4110. 10.1128/aac.00616-07 17724159 PMC2151431

[B66] Yang Q. E. Agouri S. R. Tyrrell J. M. Walsh T. R. (2018). Heavy metal resistance genes are associated with blaNDM-1-and blaCTX-M-15-carrying Enterobacteriaceae. *Antimicrob. Agents Chemother.* 62:e2642-17. 10.1128/AAC.02642-17 PMC5923091 29507071

[B67] Yang S. Deng W. Liu S. Yu X. Mustafa G. R. Chen S. (2020). Presence of heavy metal resistance genes in *Escherichia coli* and Salmonella isolates and analysis of resistance gene structure in E. coli E308. *J. Glob. Antimicrob. Resist.* 21 420–426. 10.1016/j.jgar.2020.01.009 32006752

[B68] Zhang J. Jin H. Hu J. Yuan Z. Shi W. Ran L. (2014). Serovars and antimicrobial resistance of non-typhoidal Salmonella from human patients in Shanghai, China, 2006–2010. *Epidemiol. Infect.* 142 826–832. 10.1017/s0950268813001659 23842508 PMC9151105

[B69] Zou L. Meng J. McDermott P. F. Wang F. Yang Q. Cao G. (2014). Presence of disinfectant resistance genes in *Escherichia coli* isolated from retail meats in the USA. *J. Antimicrob. Chemother.* 69 2644–2649. 10.1093/jac/dku197 24908046

