# Supplementary material for: Heavy Metal Resistance in Salmonella Typhimurium and Its Association With Disinfectant and Antibiotic Resistance
Source: Front Microbiol. 2021 Aug 4;12:702725. doi: 10.3389/fmicb.2021.702725 (PMC8371916; doi:10.3389/fmicb.2021.702725)
Supplement: Supplementary file 1 [file Table_1.DOCX]

| **Metal** | **Gene name** | **Primer(5’----3’)** | **Length** | **Reference** | **Reference annealing temperature** |
| --- | --- | --- | --- | --- | --- |
| As | *arsB* | GTSAARCCSTTYTCGATGGC  GCRAASGCSAHSAYCATGAT | 226 | Roosa et al.(2014) | 59 |
| Hg | *merA* | GAGATCTAAAGCACGCTAAGGC  GGAATCTTGACTGTGATCGGG | 1011 | Misra et al. (1984)) | 57 |
| Ni,Cr,Cd | *nccA* | ACGCCGGACATCACGAACAAG  CCAGCGCACCGAGACTCATCA | 1141 | Abou-Shanab et al. (2007) | 57 |
| Pb | *pbrA* | ATGAGCGAATGTGGCTCGAAG  TCATCGACGCAACAGCCTCAA | 2396 | Borremans et al.(2001) |  |
| Cu | *pcoA* | CGTCTCGACGAACTTTCCTG  GGACTTCACGAAACATTCCC | 1791 | Trajanovska et al.(1997) | 61 |
| Cu | *pcoC* | TTCTTACAGGTGGCCTCGTT  CCGGTAATAGGGTGCGTATC | 333 | Badar et al.(2014) |  |
| Cu | *pcoR* | CAGGTCGTTACCTGCAGCAG  CTCTGATCTCCAGGACATATC | 636 | Trajanovska et al.(1997) | 57 |
| Cr,Zn,Cd | *czcB* | CTATTTCGAACAAACAAAAGG  CTTCAGAACAAAACTGTTGG | 1520 | Nies et al.(1989) | 57 |
| Cr,Zn,Cd | *czcD* | CAGGTCACTGACACGACCAT  CATGCTGATGAGATTGATGATC | 398 | Nies et al.(1989) | 57 |
| Cr | *chrB* | GTCGTTAGCTTGCCAACATC  CGGAAAGCAAGATGTCGATCG | 450 | Nies et al.(1990) | 61 |
| Co,Ni | *cnrA* | CCTACGATCTCGCAGGTGAC  GCAGTGTCACGGAAACAACC | 422 | Romero et al.(2016) |  |
| Cd | *cadD* | AATTGCAAGTTGTGGTGCAG  CCCACACCAGGAATTCTAGC | 155 | Argudín et al.( 2016) |  |

|  |
| --- |

**Table S1: PCR primers for the detection of HMRGs**

1. Misra, T.K., Brown, N., Fritzinger, D.C., Pridmore, R.D., Barnes, W.M.,Haberstroh, L., Silver, S., 1984. Mercuric ion-resistance operons of plasmid R100 and transposon Tn501: the beginning of the operon including the regulatory region and the ﬁrst two structural genes. Proc. Nat1. Acad. Sci. USA 81, 5975–5979.
2. Nies, A., Nies, D.H., Silver, S., 1990. Nucleotide sequence and expression of a plasmid-encoded chromate resistance determinant from Alcaligenes eutrophus. J. Biol. Chem. 265, 5648–5653
3. Nies, D.H., Nies, A., Chu, L., Silver, S., 1989. Expression and nucleotide sequence of a plasmid-determined divalent cation eﬄux system from Alcaligenes eutrophus. Proc. Nat1. Acad. Sci. USA 86, 7351–7355.
4. Abou-Shanab R A I, Berkum P V, Angle J S. Heavy metal resistance and genotypic analysis of metal resistance genes in gram-positive and gram-negative bacteria present in Ni-rich serpentine soil and in the rhizosphere of Alyssum murale[J]. Chemosphere, 2007, 68(2):360-367.
5. Roosa S, Wattiez R, Prygiel E, et al. Bacterial metal resistance genes and metal bioavailability in contaminated sediments.[J]. Environmental Pollution, 2014, 189(12):143-151.
6. Trajanovska S, Britz M L, Bhave M. Detection of heavy metal ion resistance genes in Gram-positive and Gram-negative bacteria isolated from a lead-contaminated site[J]. Biodegradation, 1997, 8(2):113-24.
7. Fierros-Romero G, Gómez-Ramírez M, Arenas-Isaac G E, et al. Identification of Bacillus megaterium and Microbacterium liquefaciens genes involved in metal resistance and metal removal.[J]. Canadian Journal of Microbiology, 2016, 62(6).
8. Argudín M A, Butaye P. Dissemination of metal resistance genes among animal methicillin-resistant coagulase-negative Staphylococci.[J]. Research in Veterinary Science, 2016, 105:192-194.
9. Borremans B, Hobman J L, Provoost A, et al. Cloning and Functional Analysis of the pbr Lead Resistance Determinant of Ralstonia metallidurans CH34[J]. Journal of Bacteriology, 2001, 183(19):5651-8.
10. Badar U, Ahmed N, Shoeb E, et al. Identification of the pco operon in Enterobacter species isolated from contaminated soil[J]. Int. J, 2014, 2: 227-233.
