# Supplementary material for: Heavy Metal Resistance in Salmonella Typhimurium and Its Association With Disinfectant and Antibiotic Resistance
Source: Front Microbiol. 2021 Aug 4;12:702725. doi: 10.3389/fmicb.2021.702725 (PMC8371916; doi:10.3389/fmicb.2021.702725)
Supplement: Supplementary file 2 [file Table_2.DOCX]

Table S2: The combinations of HMRGs in 300 *Salmonella* Typhimurium strains isolated from pig meat, pig manure, poultry manure, chicken meat and human stool samples.

The combinations of HMRGs in *Salmonella Typhimurium*

Number (percentage %)

Combinations of HMRGs Overall pig meat pig manure Poultry manure Chicken meat Human stool

(N = 300) (N = 182) (N = 23) (N = 27) (N = 30) (N = 38)

czcD 20 (6.7) 9 (4.9) 0 (0) 3 (11.1) 1 (3.3) 7 (18.4)

czcD, pcoR 11 (3.7) 5 (2.7) 0 (0) 3 (11.1) 0 (0) 3 (7.9)

cnrA,pcoC,cadD,czcD,pcoR 9 (3) 6 (3.3) 2 (8.7) 1 (3.7) 0 (0) 0 (0)

nccA,czcD 9 (3) 3 (1.6) 1 (4.3) 2 (7.4) 1 (3.3) 2 (5.2)

pcoC,czcD 9 (3) 7 (3.8) 0 (0) 1 (3.3) 1 (3.7) 0 (0)

cnrA,nccA,pcoC,cadD,czcD,pcoR 8 (2.7) 5 (2.7) 1 (4.3) 1 (3.7) 0 (0) 1 (2.6)

cnrA,pcoC,czcB,pcoR 8 (2.7) 2 (1.1) 2 (8.7) 3 (11.1) 1 (3.3) 0 (0)

cnrA,nccA,pbrA,pcoC,pcoA,czdD,czcD,pcoR,merA 7 (2.3) 7 (3.8) 0 (0) 0 (0) 0 (0) 0 (0)

nccA,pbrA,pcoC,pcoA,cadD,czcD,pcoR,merA 5 (1.7) 5 (2.7) 0 (0) 0 (0) 0 (0) 0 (0)

nccA, pcoC, cadD, czcD, pcoR, merA 5 (1.7) 5 (2.7) 0 (0) 0 (0) 0 (0) 0 (0)

czcD, merA 5 (1.7) 2 (1.1) 0 (0) 0 (0) 1 (3.3) 2 (5.2)

cnrA, nccA, pbrA,pcoC, pcoA, cadD, pcoR, merA 4 (1.33) 4 (2.2) 0 (0) 0 (0) 0 (0) 0 (0)

cnrA, nccA, pbrA, pcoC,pcoA,cadD,czcD,pcoR 4 (1.33) 3 (1.6) 1 (4.3) 0 (0) 0 (0) 0 (0)

cnrA, nccA, pcoC, cadD, czcD, pcoR, merA 4 (1.33) 3 (1.6) 1 (4.3) 0 (0) 0 (0) 0 (0)

cnrA, nccA, pbrA, pcoC, czcD, pcoR, merA 4 (1.33) 2 (1.1) 1 (4.3) 1 (3.7) 0 (0) 0 (0)

cnrA, nccA, pcoC, czcD, pcoR, merA 4 (1.33) 2 (1.1) 0 (0) 0 (0) 1 (3.3) 1 (2.6)

cnrA, nccA, cadD, czcD, pcoR 4 (1.33) 0 (0) 1 (4.3) 0 (0) 3 (10) 0 (0)

nccA, pcoC, czcD, pcoR 4 (1.33) 2 (1.1) 0 (0 ) 1 (3.7) 1 (3.3) 0 (0)

pcoC, cadD, czcD, pcoR 4 (1.33) 4 (2.2) 0 (0) 0 (0) 0 (0) 0 (0)

pcoC, czcD,merA 4 (1.33) 4 (2.2) 0 (0) 0 (0) 0 (0) 0 (0)

cnrA, nccA, pcoC, czcD 4 (1.33) 4 (2.2) 0 (0) 0 (0) 0 (0) 0 (0)

cnrA, czcD,pcoR 4 (1.33) 3 (1.6) 0 (0) 0 (0) 1 (3.3) 0 (0)

pcoC, czcD,pcoR 4 (1.33) 3 (1.6) 0 (0) 1 (3.7) 0 (0) 0 (0)

cnrA, nccA,pbrA, pcoC, pcoA, czcD, pcoR, merA 3 (1) 3 (1.6) 0 (0) 0 (0) 0 (0) 0 (0)

cnrA, nccA,pbrA, pcoC,cadD, czcD, pcoR 3 (1) 1 (0.5) 0 (0) 0 (0) 2 (6.7) 0 (0)

cnrA, nccA, czcD, pcoR 3 (1) 0 (0) 0 (0) 0 (0) 3 (10) 0 (0)

cadD, pcoR,arsB 3 (1) 3 (1.6) 0 (0) 0 (0) 0 (0) 0 (0)

cadD, czcD 3 (1) 1 (0.5) 0 (0) 0 (0) 0 (0) 2 (5.3)

cnrA, czcD 3 (1) 3 (1.6) 0 (0) 0 (0) 0 (0) 0 (0)

cnrA, nccA, pcoC, czcD, pcoR 2 (0.7) 1 (0.5) 0 (0) 0 (0) 1 (3.3) 0 (0)

pcoC, pcoA, cadD,czcD, pcoR 2 (0.7) 1 (0.5) 0 (0) 0 (0) 1 (3.3) 0 (0)

cnrA, nccA, pbrA, cadD, czcD, pcoR, merA 2 (0.7) 0 (0) 1 (4.3) 0 (0) 1 (3.3) 0 (0)

cnrA, nccA, pcoC, pcoA, cadD, czcD, pcoR 2 (0.7) 2 (1.1) 0 (0) 0 (0) 0 (0) 0 (0)

nccA, pbrA,pcoC, pcoA,cadD, pcoR, merA 2 (0.7) 2 (1.1) 0 (0) 0 (0) 0 (0) 0 (0)

nccA, pcoC, pcoA, cadD,czcD, pcoR, merA 2 (0.7) 1 (0.5) 1 (4.3) 0 (0) 0 (0) 0 (0)

nccA, pcoC, pcoA, cadD, pcoR, merA 2 (0.7) 2 (1.1) 0 (0) 0 (0) 0 (0) 0 (0)

cnrA, pcoC, cadD, czcD, pcoR, merA 2 (0.7) 1 (0.5) 0 (0) 1 (3.7) 0 (0) 0 (0)

cnrA, nccA, pcoC, pcoA, cadD, czcD, czcB, pcoR 2 (0.7) 2 (1.1) 0 (0) 0 (0) 0 (0) 0 (0)

cnrA, nccA, pbrA, pcoC, pcoA, cadD, pcoR 2 (0.7) 2 (1.1) 0 (0) 0 (0) 0 (0) 0 (0)

cnrA, nccA,pbrA, cadD,czcD, pcoR 2 (0.7) 1 (0.5) 0 (0) 0 (0) 1 (3.3) 0 (0)

cnrA, nccA, cadD, czcD, pcoR, merA 2 (0.7) 1 (0.5) 0 (0) 0 (0) 1 (3.3) 0 (0)

cnrA, pcoC, czcD, pcoR, merA 2 (0.7) 2 (1.1) 0 (0) 0 (0) 0 (0) 0 (0)

nccA, pcoC, czcD, pcoR, merA 2 (0.7) 2 (1.1) 0 (0) 0 (0) 0 (0) 0 (0)

cnrA, nccA, pcoC, czcD, czcB 2 (0.7) 2 (1.1) 0 (0) 0 (0) 0 (0) 0 (0)

nccA, czcD, pcoR,merA 2 (0.7) 0 (0) 0 (0) 0 (0) 0 (0) 2 (5.3)

cnrA, cadD, czcD, pcoR 2 (0.7) 1 (0.5) 0 (0) 1 (3.7) 0 (0) 0 (0)

nccA, pcoC, cadD, czcD 2 (0.7) 2 (1.1) 0 (0) 0 (0) 0 (0) 0 (0)

nccA, pcoC, pcoA, pcoR 2 (0.7) 1 (0.5) 1 (4.3) 0 (0) 0 (0) 0 (0)

cnrA, pcoC, pcoR 2 (0.7) 0 (0) 0 (0) 0 (0) 1 (3.3) 1 (2.6)

nccA,czcD, merA 2 (0.7) 1 (0.5) 0 (0) 0 (0) 1 (3.3) 0 (0)

nccA,cadD, czcD 2 (0.7) 1 (0.5) 0 (0) 0 (0) 1 (3.3) 0 (0)

cnrA, cadD, czcD 2 (0.7) 0 (0) 1 (4.3) 0 (0) 0 (0) 1 (2.6)

nccA, pcoC, czcD 2 (0.7) 2 (1.1) 0 (0) 0 (0) 0 (0) 0 (0)

merA 2 (0.7) 1 (0.5) 0 (0) 0 (0) 0 (0) 1 (2.6)

pcoR 2 (0.7) 0 (0) 0 (0) 0 (0) 0 (0) 2 (5.2)

cnrA, nccA,pbrA, pcoC,pcoA,chrB,cadD, czcD,pcoR,merA 1 (0.3) 1 (0.5) 0 (0) 0 (0) 0 (0) 0 (0)

cnrA, nccA, pcoC, pcoA, chrB,cadD, czcD,pcoR,merA 1 (0.3) 1 (0.5) 0 (0) 0 (0) 0 (0) 0 (0)

cnrA, nccA, pcoC, pcoA, chrB,cadD, czcD,pcoR,merA 1 (0.3) 1 (0.5) 0 (0) 0 (0) 0 (0) 0 (0)

cnrA, nccA, pbrA, pcoC, chrB,cadD, czcD,pcoR,merA 1 (0.3) 0 (0) 1 (4.3) 0 (0) 0 (0) 0 (0)

cnrA, nccA, pbrA, pcoC,cadD, czcD,pcoR,merA 1 (0.3) 0 (0) 1 (4.3) 0 (0) 0 (0) 0 (0)

nccA, pcoC, pcoA, cadD, czcD,pcoR,merA 1 (0.3) 1 (0.5) 0 (0) 0 (0) 0 (0) 0 (0)

cnrA, pbrA, pcoC, pcoA, cadD,czcD,pcoR,arsB 1 (0.3) 1 (0.5) 0 (0) 0 (0) 0 (0) 0 (0)

cnrA, nccA, pbrA, pcoC, pcoA, chrB,cadD, pcoR 1 (0.3) 1 (0.5) 0 (0) 0 (0) 0 (0) 0 (0)

cnrA, nccA, pcoC, pcoA, cadD, czcD,pcoR,merA 1 (0.3) 1 (0.5) 0 (0) 0 (0) 0 (0) 0 (0)

cnrA, nccA, pbrA, pcoC, pcoA, czcD,pcoR, arsB 1 (0.3) 1 (0.5) 0 (0) 0 (0) 0 (0) 0 (0)

cnrA, nccA, pcoC, pcoA, cadD,czcD,czcB, pcoR 1 (0.3) 1 (0.5) 0 (0) 0 (0) 0 (0) 0 (0)

cnrA, nccA, pcoC, chrB,cadD, czcD,pcoR,merA 1 (0.3) 1 (0.5) 0 (0) 0 (0) 0 (0) 0 (0)

cnrA, nccA, pbrA, chrB,cadD, czcD,pcoR, arsB 1 (0.3) 0 (0) 0 (0) 0 (0) 1 (3.3) 0 (0)

nccA, pbrA, pcoC, cadD, czcD,pcoR, arsB, merA 1 (0.3) 0 (0) 0 (0) 0 (0) 1 (3.3) 0 (0)

cnrA, nccA,pcoC, cadD, czcD,czcB, pcoR 1 (0.3) 0 (0) 1 (4.3) 0 (0) 0 (0) 0 (0)

cnrA, pbrA, pcoC, pcoA, cadD, pcoR, merA 1 (0.3) 1 (0.5) 0 (0) 0 (0) 0 (0) 0 (0)

cnrA, pbrA, pcoC, cadD, czcD,pcoR,merA 1 (0.3) 1 (0.5) 0 (0) 0 (0) 0 (0) 0 (0)

cnrA, nccA, pbrA, pcoC, cadD, pcoR, merA 1 (0.3) 1 (0.5) 0 (0) 0 (0) 0 (0) 0 (0)

cnrA, nccA, pbrA, pcoC, pcoA, czcD,pcoR 1 (0.3) 1 (0.5) 0 (0) 0 (0) 0 (0) 0 (0)

nccA, pcoC, pcoA, chrB,cadD, czcD,pcoR 1 (0.3) 1 (0.5) 0 (0) 0 (0) 0 (0) 0 (0)

nccA, pcoC, chrB,cadD, czcD,pcoR,merA 1 (0.3) 1 (0.5) 0 (0) 0 (0) 0 (0) 0 (0)

cnrA, pcoC, pcoA, cadD, czcD,pcoR,merA 1 (0.3) 1 (0.5) 0 (0) 0 (0) 0 (0) 0 (0)

cnrA, pcoC, chrB,cadD, czcD,pcoR,merA 1 (0.3) 1 (0.5) 0 (0) 0 (0) 0 (0) 0 (0)

cnrA, nccA, pcoC, chrB,cadD, czcD,pcoR 1 (0.3) 1 (0.5) 0 (0) 0 (0) 0 (0) 0 (0)

cnrA, nccA, pbrA, cadD, pcoR, merA 1 (0.3) 0 (0) 1 (4.3) 0 (0) 0 (0) 0 (0)

cnrA, pbrA, cadD, czcD,pcoR,merA 1 (0.3) 0 (0) 1 (4.3) 0 (0) 0 (0) 0 (0)

cnrA, pbrA, pcoC, pcoA, czcD,pcoR 1 (0.3) 0 (0) 1 (4.3) 0 (0) 0 (0) 0 (0)

cnrA, nccA, pcoC, pcoA, cadD, pcoR 1 (0.3) 0 (0) 1 (4.3) 0 (0) 0 (0) 0 (0)

cnrA, nccA, czcD,pcoR,merA 1 (0.3) 0 (0) 1 (4.3) 0 (0) 0 (0) 0 (0)

cnrA, pcoC, czcD,czcB, pcoR 1 (0.3) 0 (0) 1 (4.3) 0 (0) 0 (0) 0 (0)

cnrA, pbrA, pcoC, czcD,pcoR,merA 1 (0.3) 0 (0) 0 (0) 1 (3.7) 0 (0) 0 (0)

cnrA, nccA, pbrA, pcoC, pcoA, pcoR, merA 1 (0.3) 0 (0) 0 (0) 0 (0) 1 (3.3) 0 (0)

cnrA, nccA, pbrA, pcoA, czcD,pcoR 1 (0.3) 0 (0) 0 (0) 0 (0) 1 (3.3) 0 (0)

cnrA, nccA, pcoC, cadD, czcD, merA 1 (0.3) 0 (0) 0 (0) 0 (0) 0 (0) 1 (2.6)

cnrA, nccA, chrB, czcD,czcB, pcoR 1 (0.3) 0 (0) 0 (0) 0 (0) 0 (0) 1 (2.6)

cnrA, nccA, pbrA, pcoC, pcoA, cadD 1 (0.3) 1 (0.5) 0 (0) 0 (0) 0 (0) 0 (0)

cadD, czcD,pcoR, arsB, merA 1 (0.3) 0 (0) 0 (0) 1 (3.7) 0 (0) 0 (0)

cnrA, czcD,pcoR, arsB, merA 1 (0.3) 0 (0) 0 (0) 1 (3.7) 0 (0) 0 (0)

nccA, pcoC, czcD,czcB, pcoR, merA 1 (0.3) 1 (0.5) 0 (0) 0 (0) 0 (0) 0 (0)

cnrA, pcoC, cadD, czcD, arsB, merA 1 (0.3) 1 (0.5) 0 (0) 0 (0) 0 (0) 0 (0)

cnrA, pcoC, pcoA, cadD, pcoR, merA 1 (0.3) 1 (0.5) 0 (0) 0 (0) 0 (0) 0 (0)

nccA, pbrA, pcoA, cadD, pcoR, merA 1 (0.3) 1 (0.5) 0 (0) 0 (0) 0 (0) 0 (0)

nccA, pbrA, pcoC, pcoA, cadD, pcoR 1 (0.3) 1 (0.5) 0 (0) 0 (0) 0 (0) 0 (0)

cnrA, pbrA, pcoC, cadD, czcD,pcoR 1 (0.3) 1 (0.5) 0 (0) 0 (0) 0 (0) 0 (0)

cnrA, nccA, chrB,cadD, czcD, merA 1 (0.3) 1 (0.5) 0 (0) 0 (0) 0 (0) 0 (0)

cnrA, nccA, pcoC, cadD, czcD,czcB 1 (0.3) 1 (0.5) 0 (0) 0 (0) 0 (0) 0 (0)

cnrA, nccA, pbrA, pcoC, czcD,pcoR 1 (0.3) 1 (0.5) 0 (0) 0 (0) 0 (0) 0 (0)

cnrA, nccA, pcoC, pcoA, pcoR 1 (0.3) 0 (0) 0 (0) 1 (3.7) 0 (0) 0 (0)

nccA, pcoC, czcD,czcB, pcoR 1 (0.3) 1 (0.5) 0 (0) 0 (0) 0 (0) 0 (0)

nccA, pcoC, cadD, pcoR, merA 1 (0.3) 1 (0.5) 0 (0) 0 (0) 0 (0) 0 (0)

nccA, cadD, czcD,czcB, merA 1 (0.3) 1 (0.5) 0 (0) 0 (0) 0 (0) 0 (0)

cnrA, pcoC, cadD, czcD, arsB 1 (0.3) 1 (0.5) 0 (0) 0 (0) 0 (0) 0 (0)

cnrA, pcoC, pcoA, chrB, pcoR 1 (0.3) 1 (0.5) 0 (0) 0 (0) 0 (0) 0 (0)

cnrA, nccA, pbrA, cadD, pcoR 1 (0.3) 0 (0) 0 (0) 0 (0) 1 (3.3) 0 (0)

nccA, cadD, czcD, arsB 1 (0.3) 0 (0) 0 (0) 0 (0) 0 (0) 1 (2.6)

czcD,pcoR, arsB, merA 1 (0.3) 1 (0.5) 0 (0) 0 (0) 0 (0) 0 (0)

nccA,chrB, czcD, merA 1 (0.3) 1 (0.5) 0 (0) 0 (0) 0 (0) 0 (0)

cnrA, cadD, czcD, merA 1 (0.3) 1 (0.5) 0 (0) 0 (0) 0 (0) 0 (0)

cnrA,pbrA, czcD. pcoR 1 (0.3) 1 (0.5) 0 (0) 0 (0) 0 (0) 0 (0)

nccA, pcoC, czcD,merA 1 (0.3) 1 (0.5) 0 (0) 0 (0) 0 (0) 0 (0)

nccA, pcoC, czcD,czcB 1 (0.3) 1 (0.5) 0 (0) 0 (0) 0 (0) 0 (0)

cnrA, nccA, cadD, czcD 1 (0.3) 0 (0) 0 (0) 0 (0) 0 (0) 1 (2.6)

cnrA, pcoC,cadD,pcoR 1 (0.3) 0 (0) 0 (0) 0 (0) 1 (0) 0 (0)

pcoC,czcD,pcoR,merA 1 (0.3) 1 (0.5) 0 (0) 0 (0) 0 (0) 0 (0)

czcD,pcoR, merA 1 (0.3) 1 (0.5) 0 (0) 0 (0) 0 (0) 0 (0)

nccA, pcoR, merA 1 (0.3) 1 (0.5) 0 (0) 0 (0) 0 (0) 0 (0)

nccA, pcoC,merA 1 (0.3) 1 (0.5) 0 (0) 0 (0) 0 (0) 0 (0)

cnrA, pcoC, czcD 1 (0.3) 1 (0.5) 0 (0) 0 (0) 0 (0) 0 (0)

pcoC, czcD,czcB 1 (0.3) 1 (0.5) 0 (0) 0 (0) 0 (0) 0 (0)

cnrA,nccA, czcD 1 (0.3) 0 (0) 0 (0) 1 (3.7) 0 (0) 0 (0)

cnrA, czcD, czcB 1 (0.3) 0 (0) 0 (0) 1 (3.7) 0 (0) 0 (0)

chrB,cadD,czcD 1 (0.3) 0 (0) 0 (0) 0 (0) 0 (0) 1 (2.6)

nccA,cadD,czcB 1 (0.3) 0 (0) 0 (0) 0 (0) 0 (0) 1 (2.6)

pcoR, merA 1 (0.3) 0 (0) 0 (0) 0 (0) 0 (0) 1 (2.6)

czcD,arsB 1 (0.3) 1 (0.5) 0 (0) 0 (0) 0 (0) 0 (0)

pcoC,merA 1 (0.3) 1 (0.5) 0 (0) 0 (0) 0 (0) 0 (0)

czcD, czcB 1 (0.3) 0 (0) 0 (0) 1 (3.7) 0 (0) 0 (0)

cnrA 1 (0.3) 1 (0.5) 0 (0) 0 (0) 0 (0) 0 (0)
