# Supplementary material for: Heavy Metal Resistance in Salmonella Typhimurium and Its Association With Disinfectant and Antibiotic Resistance
Source: Front Microbiol. 2021 Aug 4;12:702725. doi: 10.3389/fmicb.2021.702725 (PMC8371916; doi:10.3389/fmicb.2021.702725)
Supplement: Supplementary file 3 [file Table_3.docx]

**Table S3:** The combinations of antibiotic resistance in 300 *Salmonella* Typhimurium strains isolated from pig meat, pig manure, poultry manure, chicken meat and human stool samples.

**The combinations of Antibiotic Resistance in *Salmonella* Typhimurium**

Number (percentage %)

Combinations of Antibiotics Overall pig meat pig manure Poultry manure chicken meat Human stool

(N = 300) (N = 182) (N = 23) (N = 30) (N = 27) (N = 38)

TET,AMP,SXT,C,NA,TMP,CN,S3,S 36 (12) 31 (17) 3 (13) 0 (0) 0 (0) 2 (5.3)

TET,AMP,SXT,C,TMP,S3,S 25 (8.3) 20 (11) 4 (17.4) 0 (0) 0 (0) 1 (2.6)

TET,AMC,AMP,SXT,C,NA,TMP,CN,S3,S 23 (7.6) 21 (11.5) 0 (0) 0 (0) 2 (6.7) 0 (0)

NA 19 (6.3) 1 (0.5) 0 (0) 2 (7.4) 9 (30) 7 (18.4)

TET,AMP,S3,S 16 (5.3) 7 (3.8) 0 (0) 0 (0) 1 (3.3) 8 (21)

NA,S 13 (4.3) 5 (2.7) 0 (0) 2 (7.4) 4 (13.3) 2 (5.2)

TET,AMP,NA,S3,S 12 (4) 9 (4.9) 0 (0) 0 (0) 0 (0) 3 (7.9)

TET,AMP,SXT,NA,TMP,CN,S3,S 11 (3.6) 11 (6) 0 (0) 0 (0) 0 (0) 0 (0)

TET,AMC,AMP,SXT,CIP,C,OFX,NA,TMP,CN,S3,S 10 (3.3) 4 (2.2) 6 (26) 0 (0) 0 (0) 0 (0)

S 9 (3) 3 (1.6) 0 (0) 5 (18.5) 0 (0) 1 (2.6)

TET,AMP,SXT,C,TMP,CN,S3,S 6 (2) 6 (3.3) 0 (0) 0 (0) 0 (0) 0 (0)

TET,AMP,C,NA,S3,S 6 (2) 4 (2.2) 0 (0) 2 (7.4) 0 (0) 0 (0)

TET,SXT,C,TMP,S3,S 6 (2) 1 (0.5) 5 (21.7) 0 (0) 0 (0) 0 (0)

TET,AMP,SXT,C,NA,TMP,S3,S 5 (1.7) 3 (1.6) 1 (4.3) 1 (3.7) 0 (0) 0 (0)

AMP,SXT,C,TMP,S3,S 5 (1.7) 4 (2.2) 0 (0) 0 (0) 1 (3.3) 0 (0)

TET,AMP,SXT,C,TMP,S3 5 (1.7) 3 (1.6) 2 (8.7) 0 (0) 0 (0) 0 (0)

TET,AMP,SXT,C,OFX,NA,TMP,CN,S3,S 4 (1.3) 4 (2.2) 0 (0 ) 0 (0) 0 (0) 0 (0)

TET,AMP,C,NA,CN,S3,S 4 (1.33) 0 (0) 0 (0) 3 (11.1) 1 (3.3) 0 (0)

TET,AMP,SXT,C,NA,TMP,S3 3 (1.33) 3 (1.6) 0 (0) 0 (0) 0 (0) 0 (0)

TET,AMP,NA,S 3 (1.33) 0 (0) 0 (0) 0 (0) 0 (0) 3 (7.9)

TET,AMP,SXT,CIP,C,NA,TMP,S3,S 2 (0.7) 2 (1.1) 0 (0) 0 (0) 0 (0) 0 (0)

TET,AMC,AMP,C,NA,CN,S3,S 2 (0.7) 2 (1.1) 0 (0) 0 (0) 0 (0) 0 (0)

TET,AMP,SXT,C,NA,CN,S3,S 2 (0.7) 2 (1.1) 0 (0) 0 (0) 0 (0) 0 (0)

TET,AMP,SXT,C,CN,S3,S 2 (0.7) 2 (1.1) 0 (0) 0 (0) 0 (0) 0 (0)

NA,S3,S 2 (0.7) 1 (0.5) 0 (0) 0 (0) 0 (0) 1 (2.6)

TET,S3,S 2 (0.7) 1 (0.5) 0 (0) 1 (3.7) 0 (0) 0 (0)

TET,NA 2 (0.7) 2 (1.1) 0 (0) 0 (0) 0 (0) 0 (0)

S3 2 (0.7) 0 (0) 0 (0) 0 (0) 2 (6.7) 0 (0)

AMC 2 (0.7) 0 (0) 0 (0) 2 (7.4) 0 (0) 0 (0)

TET,AMC,AMP,SXT,CIP,C,OFX,NA,TMP,CAZ,CN,S3,S 1 (0.3) 0 (0) 1 (4.3) 0 (0) 0 (0) 0 (0)

TET,AMP,SXT,CIP,C,OFX,NA,TMP,CN,S3,S 1 (0.3) 1 (0.5) 0 (0) 0 (0) 0 (0) 0 (0)

TET,AMC,AMP,SXT,CIP,C,OFX,NA,TMP,S3,S 1 (0.3) 0 (0) 0 (0) 0 (0) 1 (3.3) 0 (0)

TET,AMC,AMP,SXT,C,OFX,NA,TMP,CN,S3,S 1 (0.3) 1 (0.5) 0 (0) 0 (0) 0 (0) 0 (0)

TET,AMP,SXT,CIP,C,NA,TMP,CN,S3,S 1 (0.3) 1 (0.5) 0 (0) 0 (0) 0 (0) 0 (0)

TET,AMP,SXT,CIP,C,OFX,NA,TMP,S3,S 1 (0.3) 0 (0) 0 (0) 0 (0) 1 (3.3) 0 (0)

TET,AMC,AMP,CIP,OFX,CTX,TMP,CN,S3,S 1 (0.3) 0 (0) 0 (0) 0 (0) 0 (0) 1 (2.6)

AMP,SXT,C,NA,TMP,CN,S3,S 1 (0.3) 0 (0) 0 (0) 0 (0) 0 (0) 1 (2.6)

TET,AMC,AMP,SXT,C,CTX,TMP,CAZ,S3,S 1 (0.3) 0 (0) 0 (0) 0 (0) 0 (0) 1 (2.6)

TET,AMC,AMP,SXT,C,NA,CN,S3,S 1 (0.3) 0 (0) 0 (0) 0 (0) 1 (3.3) 0 (0)

TET,AMP,SXT,CIP,C,NA,TMP,CN,S3 1 (0.3) 1 (0.5) 0 (0) 0 (0) 0 (0) 0 (0)

TET,AMC,AMP,SXT,C,NA,TMP,S3,S 1 (0.3) 1 (0.5) 0 (0) 0 (0) 0 (0) 0 (0)

TET,AMP,SXT,CIP,NA,TMP,CN,S3,S 1 (0.3) 0 (0) 0 (0) 0 (0) 0 (0) 1 (2.6)

TET,AMC,AMP,SXT,C,TMP,CAZ,S3,S 1 (0.3) 0 (0) 0 (0) 0 (0) 0 (0) 1 (2.6)

AMC,AMP,SXT,C,NA,TMP,CN,S3,S 1 (0.3) 1 (0.5) 0 (0) 0 (0) 0 (0) 0 (0)

TET,AMC,AMP,CIP,C,NA,TMP,S3,S 1 (0.3) 1 (05) 0 (0) 0 (0) 0 (0) 0 (0)

TET,AMC,AMP,CIP,C,OFX,NA,S3,S 1 (0.3) 1 (0.5) 0 (0) 0 (0) 1 (3.3) 0 (0)

TET,AMP,SXT,NA,TMP,CAZ,CN,S3 1 (0.3) 1 (0.5) 0 (0) 0 (0) 0 (0) 0 (0)

AMP,SXT,CIP,C,NA,TMP,CN,S3 1 (0.3) 1 (0.5) 0 (0) 0 (0) 0 (0) 0 (0)

TET,AMC,SXT,C,NA,CN,S3,S 1 (0.3) 1 (0.5) 0 (0) 0 (0) 0 (0) 0 (0)

TET,AMP,SXT,CIP,C,NA,S3,S 1 (0.3) 1 (0.5) 0 (0) 0 (0) 0 (0) 0 (0)

AMP,SXT,C,NA,TMP,CN,S3,S 1 (0.3) 1 (0.5) 0 (0) 0 (0) 0 (0) 0 (0)

AMP,SXT,C,NA,TMP,CN,S3 1 (0.3) 0 (0) 0 (0) 0 (0) 0 (0) 1 (2.6)

TET,SXT,C,NA,TMP,CN,S3 1 (0.3) 1 (0.5) 0 (0) 0 (0) 0 (0) 0 (0)

TET,SXT,C,NA,TMP,CN,S3,S 1 (0.3) 0 (0) 1 (4.3) 0 (0) 0 (0) 0 (0)

TET,AMP,SXT,NA,CN,S3,S 1 (0.3) 1 (0.5) 0 (0) 0 (0) 0 (0) 0 (0)

TET,AMC,AMP,SXT,CN,S3,S 1 (0.3) 1 (0.5) 0 (0) 0 (0) 0 (0) 0 (0)

TET,AMC,AMP,C,S3,S 1 (0.3) 1 (0.5) 0 (0) 0 (0) 0 (0) 0 ()

TET,AMC,SXT,C,TMP,S3 1 (0.3) 0 (0) 0 (0) 1 (3.7) 0 (0) 0 (0)

TET,AMP,C,NA,CN,S3 1 (0.3) 0 (0) 0 (0) 0 (0) 0 (0) 1 (2.6)

TET,AMP,C,CN,S3,S 1 (0.3) 0 (0) 0 (0) 0 (0) 1 (3.3) 0 (0)

TET,AMP,SXT,CN,S3,S 1 (0.3) 1 (0.5) 0 (0) 0 (0) 0 (0) 0 (0)

TET,AMP,C,S3,S 1 (0.3) 1 (0.5) 0 (0) 0 (0) 0 (0) 0 (0)

TET,C,NA,S3,S 1 (0.3) 1 (0.5) 0 (0) 0 (0) 0 (0) 0 (0)

TET,AMC,AMP,NA,S 1 (0.3) 0 (0) 0 (0) 1 (3.7) 0 (0) 0 (0)

TET,AMC,NA,S 1 (0.3) 0 (0) 0 (0) 1 (3.7) 0 (0) 0 (0)

TET,AMP,NA,S3 1 (0.3) 1 (0.5) 0 (0) 0 (0) 0 (0) 0 (0)

TET,NA,S3,S 1 (0.3) 1 (0.5) 0 (0) 0 (0) 0 (0) 0 (0)

NA,CTX,S3 1 (0.3) 0 (0) 0 (0) 0 (0) 1 (3.3) 0 (0)

TET,C,S3 1 (0.3) 0 (0) 0 (0) 0 (0) 0 (0) 1 (2.6)

TET,AMP,S3 1 (0.3) 1 (0.5) 0 (0) 0 (0) 0 (0) 0 (0)

C,S3,S 1 (0.3) 0 (0) 0 (0) 0 (0) 0 (0) 1 (2.6)

AMP,S3,S 1 (0.3) 1 (0.5) 0 (0) 0 (0) 0 (0) 0 (0)

TET,C,NA 1 (0.3) 1 (0.5) 0 (0) 0 (0) 0 (0) 0 (0)

CIP,NA,S 1 (0.3) 1 (0) 0 (0) 1 (3.7) 0 (0) 0 (0)

S3,S 1 (0.3) 0 (0) 0 (0) 0 (0) 0 (0) 1 (2.6)

TET,C 1 (0.3) 1 (0.5) 0 (0) 0 (0) 0 (0) 0 (0)

TET 1 (0.3) 1 (0.5) 0 (0) 0 (0) 0 (0) 0 (0)
